# Symbionts do not affect the mating incompatibility between the Brazilian-1 and Peruvian morphotypes of the *Anastrepha fraterculus* cryptic species complex

**DOI:** 10.1038/s41598-019-54704-y

**Published:** 2019-12-04

**Authors:** Francisco Devescovi, Claudia A. Conte, Antonios Augustinos, Elena I. Cancio Martinez, Diego F. Segura, Carlos Caceres, Silvia B. Lanzavecchia, Kostas Bourtzis

**Affiliations:** 1 Instituto de Agrobiotecnología y Biología Molecular (IABIMO-CONICET), Hurlingham, B1686 Buenos Aires, Argentina; 2 Instituto de Genética “E.A. Favret”, Instituto Nacional de Tecnología Agropecuaria, Hurlingham, B1686 Buenos Aires, Argentina; 3 Insect Pest Control Laboratory, Joint FAO/IAEA Division of Nuclear Techniques in Food and Agriculture, Vienna International Centre, P.O. Box 100, 1400 Vienna, Austria; 4 Present Address: Department of Plant Protection, Institute of Industrial and Forage Crops, Hellenic Agricultural Organization – DEMETER, Patras, Greece

**Keywords:** Agricultural genetics, Entomology

## Abstract

The South American fruit fly, *Anastrepha fraterculus*, is clearly undergoing a speciation process. Among others, two of their morphotypes, the Brazilian-1 and Peruvian, have accumulated differences in pre- and post-zygotic mechanisms resulting in a degree of reproductive isolation. Both harbor a different strain of *Wolbachia*, which is a widespread endosymbiotic bacterium among many invertebrates producing a range of reproductive effects. In this paper, we studied the role of this bacterium as one of the factors involved in such isolation process. Infected and cured laboratory colonies were used to test pre- and post-zygotic effects, with special emphasis in uni- and bi-directional cytoplasmic incompatibility (CI). We showed that *Wolbachia* is the only known reproductive symbiont present in these morphotypes. *Wolbachia* reduced the ability for embryonic development in crosses involving cured females and infected males within each morphotype (uni-directional CI). This inhibition showed to be more effective in the Peruvian morphotype. Bi-directional CI was not evidenced, suggesting the presence of compatible *Wolbachia* strains. We conclude that *Wolbachia* is not directly involved in the speciation process of these morphotypes. Other mechanisms rather than CI should be explored in order to explain the reduced mating compatibility between the Brazilian-1 and Peruvian morphotypes.

## Introduction

Throughout the history of life on earth, evolution and speciation of living organisms have been highly influenced by interactions with other species. Among these interactions, symbiosis has had major consequences, as evidenced in the origin of eukaryotic cells^1,2^. Insects are marked by many types of interactions with different microorganisms, ranging from beneficial to harmful. Among these, the association with bacteria of the *Wolbachia* genus is maybe the most widespread symbiosis that insects have established throughout their radiation and evolution^3^. *Wolbachia* includes obligate intracellular endosymbiotic bacteria that are maternally transmitted through the cytoplasm of the eggs, taking full advantage of the host to develop their life cycle^4,5^. These bacteria are phylogenetically distributed in different supergroups within the order Rickettsiales, the majority of which participate in symbiotic relationships with different groups of arthropods^3,6,7^. It was also suggested that *Wolbachia* is horizontally transmitted between hosts, as phylogenies of *Wolbachia* infecting arthropods and its hosts species are not congruent^5,8–11^.

*Wolbachia* affects the reproductive biology of its hosts in different ways, inducing reproductive alterations such as feminization of genetic males, parthenogenesis in haplodiploid species, the killing of male progeny deriving from infected females, and embryonic mortality due to cytoplasmic incompatibility (CI)^3,12,13^. In all cases, *Wolbachia* ultimately favour their vertical transmission as reproductive parasites at the expense of the uninfected host population. Among these reproductive phenotypes, CI is the most extensively studied, with a potential applied use in insect control strategies^14–16^. CI can be expressed either uni-directionally or bi-directionally^3,5^. In the former, CI typically occurs when a *Wolbachia*-infected male is crossed with an uninfected female^17–19^. The reciprocal cross (infected female and uninfected male) is fully compatible, as are crosses between infected individuals. Bi-directional CI occurs in crosses between infected individuals harbouring different and incompatible *Wolbachia* strains. In contrast, crosses between females and males infected with the same or compatible *Wolbachia* strains produce viable progeny^20^.

CI is markedly variable in intensity and pattern among host species and these characteristics are potentially involved in the promotion of a host-symbiont co-evolution^3,21^. Since CI acts as a post-zygotic isolation mechanism between populations with different infection status or harbouring incompatible *Wolbachia* strains, it was suggested that they could influence evolutionary processes of their hosts including speciation (*e.g. Drosophila*^20,22–24^, *Nasonia* complex^18,24,25^, *Gryllus* crickets^26^).

*Wolbachia* has been found in the South American fruit fly, *Anastrepha fraterculus* (Diptera: Tephritidae)^27–29^, and a potential role on the mating isolation and the speciation process within the cryptic complex was suggested^30^. *A. fraterculus* has a wide distribution ranging from Argentina to northern Mexico^31^. This species represents a threat to several fruit crops in South America^32^ and generates important economic losses due to direct damage through oviposition and larval development and quarantine restrictions for exportation^33^. Previous studies have described at least eight morphotypes for this complex, arranged along the full range of distribution, with subtle differences in their morphology and biology^34^. A large component of the observed pre-zygotic isolation between two natural populations from Peru and Argentina (the Peruvian and Brazilian-1 morphotypes, which for the purpose of this paper were named as ‘AfP’ and ‘AfC’, respectively) is due to differences in the mating time between the two parental strains^35^, although viable hybrids can be obtained^30,36^. Egg-hatch reduction and sex ratio distortion were also detected as a degree of post-zygotic isolation, probably indicative of major genetic differences as suggested by gross asynapsis in the hybrids^30^. Also, it was suggested that some type of nuclear–cytoplasmic interaction is present, although probably not associated to *Wolbachia*. Both the Peruvian and Brazilian-1 morphotypes were found singly infected by different strains (namely *w*Per and *w*Arg) of this symbiont based on the partial sequencing of the *wsp* gene^30^. Later, it was shown that the Brazilian-1 morphotype contains individuals that can harbour one of two nucleotide variants of *Wolbachia* (*w*AfraCast1_A and *w*AfraCast2_A)^37^.

Given that *Wolbachia* infections may affect life history traits of its hosts including productivity, mating behaviour, and reproduction, unravelling the role of *Wolbachia* within the *A. fraterculus* complex might help for the implementation of an effective Sterile Insect Technique (SIT) or other related techniques against these major pests. SIT is a species-specific strategy which relies on the mass release of sterilized males that will transfer inviable sperm to wild females, therefore producing inviable embryos. As the SIT is usually applied on large areas^38^, not only the boundaries of each morphotype distribution and the overlapping zones need to be considered for a cryptic species-complex like *A. fraterculus* but also their ecological interactions, which *Wolbachia* may be affecting.

In this work, we aim to determine whether the mating incompatibility observed between the Brazilian-1 and Peruvian morphotypes of the *A. fraterculus* cryptic species complex is symbiont-mediated and, in particular, whether the presence of *Wolbachia* is associated with phenomena of uni- and bi-directional CI. To this end, crossing experiments within and between morphotypes were carried out using infected and cured *A. fraterculus*, analysing a series of pre- and post-zygotic parameters associated to sexual isolation, including key variables affected by CI.

## Results

The single infection and the presence of the different *Wolbachia* strains in the studied colonies of *A. fraterculus* was confirmed, and the presence of *w*Per and *w*AfraCast2_A in AfP and AfC, respectively (Supp. Fig. 1) was verified. The sequence analysis of the *wsp* gene performed through the *Wolbachia* MLST website evidenced the presence of different *wsp* alleles and HVR allelic profiles of *Wolbachia* infecting AfC and AfP (Table 1). MLST data analysis from each colony assigned a unique sequence type (ST-13) with the allelic profile 1:1:1:3:1, corresponding to the alleles of the five genes analysed.

**Table 1:** Molecular characterization of *Wolbachia* by HVRs and *wsp* analyses.

*Wolbachia* strain ID	HVR1	HVR2	HVR3	HVR4	*wsp* allele
*w*Per	1	12	21	19	23
*w*AfraCast2_A	1	12	21	283	663

HVR alleles were assigned considering translated nucleotide sequences and *wsp* alleles were established by comparing the obtained sequences against a *Wolbachia* nucleotide database.

After the analysis of 20 individuals of each *A. fraterculus* colony, the presence of *Wolbachia* was confirmed in all individuals belonging to infected colonies (100% prevalence). In addition, the absence of an active infection of *Wolbachia* was confirmed in each *Wolbachia*-cured colony (AfP− and AfC−; Supp. Figs. 2 and 3, respectively). Additionally, after the analysis of at least fifteen DNA samples from each of the *A. fraterculus* strains studied, the absence of the reproductive symbionts *Spiroplasma* sp., *Cardinium* sp., *Rickettsia* sp., and *Arsenophonus* sp., was confirmed.

### Experiment 1: AfP crosses

#### Pre-zygotic incompatibility tests

**Figure 1:**
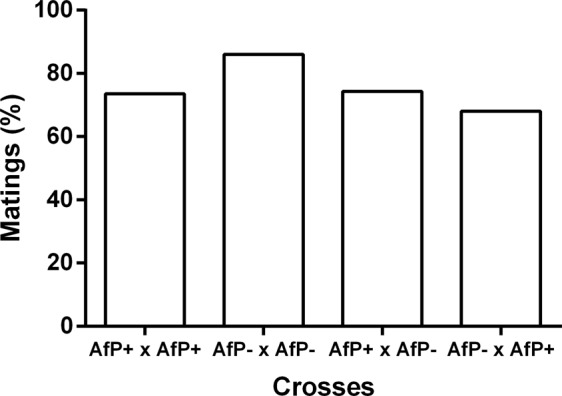
Percentage of matings recorded for each type of cross involving *Wolbachia*-cured (−) and infected (+) females and males belonging to the Peruvian morphotype of *Anastrepha fraterculus* (AfP). Cross notation: Female × male.

The four different crosses of cured and infected AfP females and males showed no significant differences in the percentage of matings (X^2^ = 4.63, d.f. = 3, N = 169, p = 0.201, Fig. 1). The mean percentage of matings (±SE) was 75.5 ± 3.8%.

The latency to mate was statistically different among treatments [F_(3, 111)_ = 7.20, p = 0.0002, Table 2]. The crossing involving AfP− females and AfP− males mated faster than the other combinations. Also, mating duration was affected by the infection status [F_(3, 117)_ = 19.29, p < 0.0001, Table 2]. Multiple contrasts showed that matings involving AfP− females ended significantly later (p < 0.001) than those involving AfP+ females, irrespectively of the male infection status.

**Table 2:** Latencies to mate and mating durations (mean ± SE, in minutes) for the different combinations of *Wolbachia*-cured (−) or infected (+) *Anastrepha fraterculus*.

	Cross	Latency	Duration
Experiment 1	AfP+ × AfP+	129.60 ± 10.57 a	25.48 ± 3.31 a
	AfP− × AfP−	72.77 ± 10.74 b	64.52 ± 6.88 b
	AfP+ × AfP−	139.40 ± 14.22 a	33.28 ± 6.07 a
	AfP− × AfP+	121.12 ± 9.34 a	63.42 ± 6.36 b
Experiment 2	AfC+ × AfC+	2.68 ± 2.68 a	53.92 ± 4.03 a
	AfC− × AfC−	7.09 ± 3.09 b	80.91 ± 5.10 c
	AfC+ × AfC−	7.29 ± 4.40 b	58.29 ± 3.62 ab
	AfC− × AfC+	2.28 ± 2.28 a	73.06 ± 4.46 bc
Experiment 3	AfP+ × AfC+	99.83 ± 19.50 ab	20.87 ± 2.47 a
	AfC+ × AfP+	122.38 ± 20.66 ab	43.38 ± 3.08 b
	AfP− × AfC−	162.9 ± 18.52 a	25.65 ± 1.76 a
	AfC− × AfP−	97.93 ± 14.22 b	50.37 ± 4.42 b

Same letters denote no significant differences among treatments after ANOVA performed for each experiment and dependent variable. Different letters denote significant differences at α = 0.05 after Tukey tests.

#### Post-zygotic incompatibility tests

**Figure 2:**
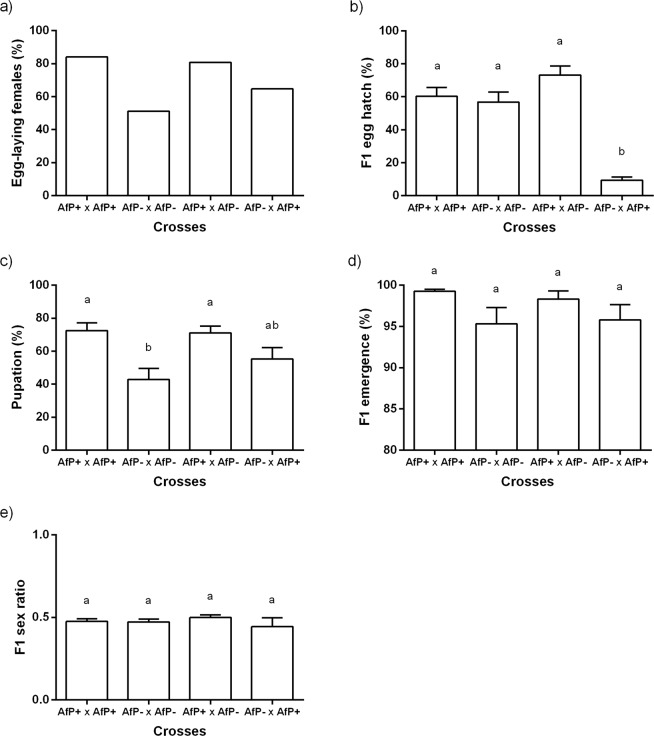
Post-zygotic variables obtained from crosses of *Wolbachia* cured (−) or infected (+) Peruvian individuals of *Anastrepha fraterculus* (AfP). (**a**) Percentage of egg-laying females out of the mated couples, (**b**) percentage of hatched eggs, (**c**) percentage of hatched larvae that reached the pupal stage, (**d**) percentage of adult emergence, and (**e**) their sex ratio. Different letters within each dependent variable denote significant differences at α = 0.05 after Tukey’s tests. Same letters denote no significant effect of the treatment. Cross notation: Female × male.

The percentage of females that laid more than 50 fertilized eggs did not differ statistically among crosses (X^2^ = 6.77, d.f. = 3, N = 128, p = 0.080, Fig. 2a), showing a general average (±S.E.) of 75.03 ± 6.17%. The percentage of egg hatch differed statistically among crosses [F_(3, 79)_ = 45.93, p < 0.0001], with lower values in the cross between AfP− females and AfP + males compared to the other crosses (Fig. 2b). The percentage of pupation was statistically different among crosses [F_(3, 67)_ = 6.45, p < 0.0007], with lower values for the cross between AfP− flies compared to the two crosses involving AfP+ (Fig. 2c). Percentage of pupation in the crosses between AfP− females and AfP+ males showed intermediate values (Fig. 2c). Adult emergence did no differ among crosses [F_(3, 54)_ = 0.58, p = 0.630, Fig. 2d], with an average emergence (±S.E.) of 97.55 ± 0.63%; as neither did the sex ratio [F_(3, 53)_ = 1.20, p = 0.319], with an almost equal production of males and females (Fig. 2e).

### Experiment 2: AfC crosses

#### Pre-zygotic incompatibility tests

**Figure 3:**
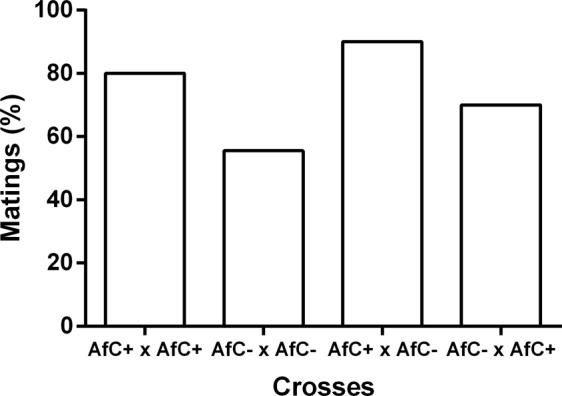
Percentage of matings between *Wolbachia*-cured (−) or infected (+) males and females belonging to the Brazilian-1 morphotype of *Anastrepha fraterculus* (AfC). Cross notation: Female × male.

There was a significant effect of the type of cross on the percentage of matings (X^2^ = 11.83, d.f. = 3, N = 135, p = 0.008, Fig. 3) mainly due to the lower values observed in the crosses involving AfC− flies when singly compared with either crosses involving AfC+ females (AfC− × AfC− vs. AfC+ × AfC−: X^2^ = 10.04, d.f. = 1, n = 75, p = 0.002; AfC− × AfC− vs. AfC+ × AfC+ : X^2^ = 4.75, d.f. = 1, n = 75, p = 0.029).

Latency to mate was statistically different among treatments [F_(3, 83) = _10.21, p = 0.00001, Table 2]. Crosses involving AfC+ males showed similar values and mated faster than those involving AfC− males. Likewise, mating duration was also significantly different among crosses [F_(3, 81)_ = 8.78, p = 0.0004], with both crosses involving AfC+ females showing the lowest values and the cross between AfC− females and males the highest (Table 2).

#### Post-zygotic incompatibility tests

**Figure 4:**
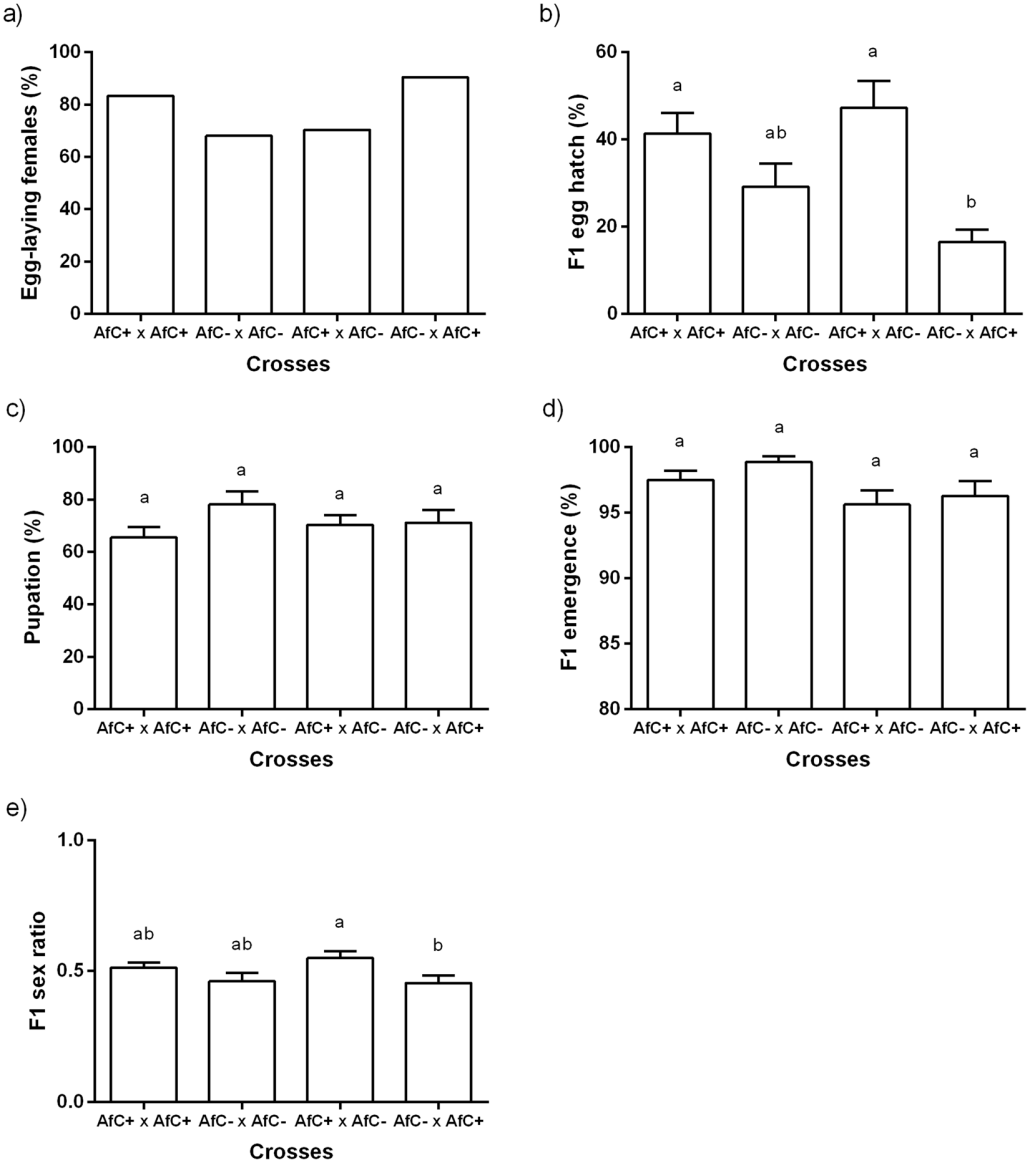
Post-zygotic variables obtained for crosses of *Wolbachia*-cured (−) or infected (+) Brazilian-1 individuals of *Anastrepha fraterculus* (AfC). (**a**) Percentage of egg-laying females out of the mated couples, (**b**) percentage of hatched eggs, (**c**) percentage of hatched larvae that reached the pupal stage, (**d**) percentage of adult emergence, and (**e**) their sex ratio. Different letters within each dependent variable denote significant differences at α = 0.05 after Tukey’s tests. Same letters denote no significant effect of the treatment. Cross notation: Female × male.

The percentage of egg-laying females was similar among treatments (X^2^ = 4.55, d.f. = 3, N = 97, p = 0.208, Fig. 4a), with a general mean value (±S.E.) of 78.04 ± 5.34%. The percentage of egg hatch was statistically different among crosses [F_(3, 69)_ = 8.05, p = 0.0001]. The lowest percentage of egg hatch was obtained from the crosses between AfC− females and AfC+ males, which differed from the two crosses involving AfC+ females (Fig. 4b). Crosses between AfC− flies produced an intermediate value, similar to the other treatments. The percentages of pupation and adult emergence did not differ statistically among crosses [% pupation: F_(3, 58)_ = 1.31, p = 0.279; % adult emergence: F_(3, 54)_ = 1.90, p = 0.140], with general mean values (±S.E.) of 70.44 ± 2.15% and 96.95 ± 0.48%, respectively (Fig. 4c,d). On the other hand, the sex ratio was statistically different among treatments [F_(3,54)_ = 3.40, p = 0.024, Fig. 4e]. Multiple comparisons showed a significant increase in the proportion of males in the cross of AfC− females and AfC+ males when compared to its reciprocal cross (p = 0.049), but similar to the cross between AfC− flies.

### Experiment 3: AfP × AfC crosses

#### Pre-zygotic incompatibility tests

**Figure 5:**
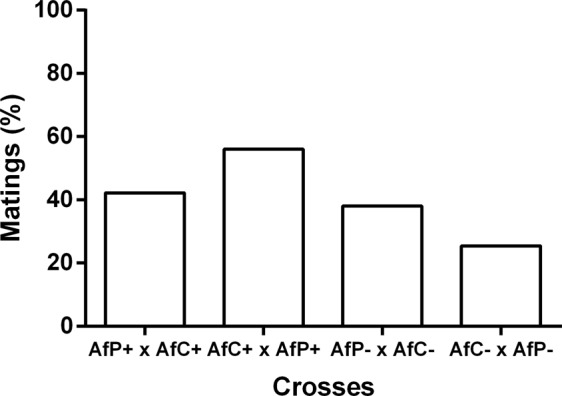
Percentage of matings from crosses between the Brazilian-1 (AfC) and Peruvian (AfP) morphotypes using *Wolbachia*-cured (−) or infected (+) males and females. Cross notation: Female × male.

The percentage of mated couples was overall low, and different among crosses (X^2^ = 14.74, d.f. = 3, N = 324, p = 0.002, Fig. 5). Pairwise comparisons showed statistically higher values in the cross of AfC+ females and AfP+ males when compared either with the same type of cross but involving cured flies (X^2^ = 14.10, d.f. = 1, n = 160, p < 0.01) and the cross of AfP− females and AfC− males (X^2^ = 4.38, d.f. = 1, n = 150, p = 0.036). Also, a higher percentage of matings was observed in the cross of AfP+ females and AfC+ males when compared with the cross of AfC− females and AfP− males (X^2^ = 5.24, d.f. = 1, n = 174, p = 0.022).

Statistical differences in the latency to mate were observed among crosses [F_(3, 121)_ = 2.99, p = 0.034]. Multiple comparisons showed that matings involving AfC− females and AfP− males occurred faster than those involving AfP− females and AfC− males (Table 2), with the remaining crosses taking intermediate values. Mating duration of the two types of crosses involving AfP females and AfC males were statistically lower than the two types of crosses involving AfC females and AfP males, independently of the infection status [F_(3, 118)_ = 23.59, p = 0.01, Table 2].

#### Post-zygotic incompatibility tests

**Figure 6:**
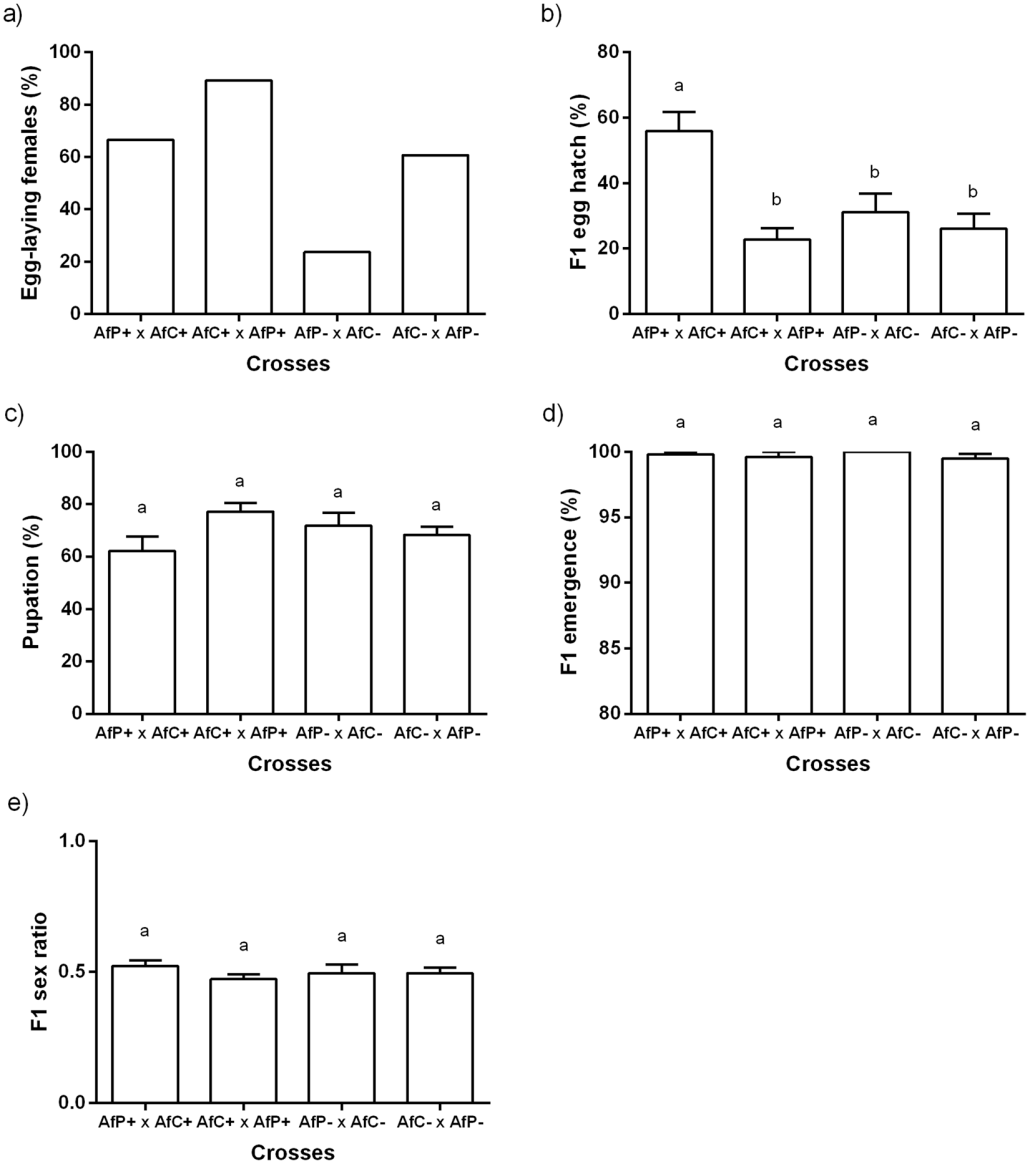
Post-zygotic variables obtained for crosses of *Wolbachia*-cured (−) or infected (+) Brazilian-1 (AfC) and Peruvian (AfP) individuals of *Anastrepha fraterculus*. (**a**) Percentage of egg-laying females out of the mated couples, (**b**) percentage of hatched eggs, (**c**) percentage of hatched larvae that reached the pupal stage, (**d**) percentage of adult emergence, and (**e**) their sex ratio. Different letters within each dependent variable denote significant differences at α = 0.05 after Tukey’s tests. Same letters denote no significant effect of the treatment. Cross notation: Female × male.

The percentage of egg-laying females was statistically different among treatments (X^2^ = 30.31, d.f. = 3, N = 121, p = 0.001, Fig. 6a), and it was lowest for the crosses between AfP− females and AfC− males. The percentage of egg hatch also showed differences among treatments [F_(3, 65)_ = 10.96, p = 0.00001]. The crosses between AfP+ females and AfC+ males showed significantly higher values when compared to all other crosses (p <0.05), which in turn did not differ among each other (Fig. 6b). Percentage of pupation [F_(3, 49)_ = 2.75, p = 0.053] and percentage of adult emergence [F_(3, 44)_ = 0.72, p = 0.545] were similar among treatments, with general mean values (±S.E.) of 71.02 ± 2.19% and 99.68 ± 0.19%, respectively (Fig. 6c,d). Likewise, no differences in the sex ratio were detected among treatments [F_(3,44)_ = 0.91, p = 0.445, Fig. 6e], with a mean value of 0.49 ± 0.01.

## Discussion

In the present study we evaluated the reproductive compatibility within and between two morphotypes belonging to the *A. fraterculus* cryptic species complex infected by single and different *Wolbachia* strains, considering the potential role of the endosymbiont in the previously observed phenotypic and behavioural differences^30,37^. Within each colony, cured females that mated with infected males had a significantly reduced egg hatch when compared to the other type of crosses. These results on this key variable suggest uni-directional CI. The absence of bi-directional CI was evidenced in crosses involving both infected morphotypes, as no egg hatch reduction was observed. Despite large similarities in the studied parameters observed among heterotypic crosses, our results suggest that the presence of *Wolbachia* might affect them in a different way. This could be a consequence of the differences between *Wolbachia* strains^29,30,37^, differences in the genetic background of the hosts^30,39^ or a distinctive interaction between each morphotype and its reproductive symbiont. Differences in the density of the symbiont within cells, which was not assessed, may have also accounted for the observed differences as previously shown in *Drosophila* species^40,41^ and the wasp *Nasonia vitripennis* (Hymenoptera: Pteromalidae)^42^. Yet, independently of the mechanism, the reduced number of matings between morphotypes was confirmed.

Based on the sequence of the *wsp* gene, different *Wolbachia* strains and 100% prevalence were detected in the *A. fraterculus* colonies used in our experiments. In the case of AfP, our results showed the presence of *w*Per strain, as was previously reported by Cáceres and his colleagues^30^. Sequence analyses showed identical MLST allelic profiles between *w*Per and the *w*AfraCast1_A strain infecting *A. fraterculus* (Brazilian-1 morphotype) from Argentina^37^. In addition, we confirmed the allelic patterns of *Wolbachia* infecting AfC (*w*AfraCast2_A) as was previously characterized by Conte *et al*.^37^.

Differences between morphotypes were observed from pre-zygotic tests. For AfP, the lack of an effect on the mating percentage suggests that the infection does not affect flies’ mating propensity for either sex. However, an infected status may represent possible fitness costs, as the latency to mate decreased and the mating duration increased when flies were cured from *Wolbachia*. These characteristics have been proposed to be advantageous^43,44^. In the case of AfC, the absence of *Wolbachia* in both sexes correlates with a lower tendency to copulate, again suggesting a negative effect associated with the curing protocol. Cured AfC males needed more time to mate and all matings involving a cured fly lasted longer, which may indicate that the curing process or the absence of some bacterial taxa (even though not checked, probably removed by the antibiotic treatment) affected the mating parameters in each morphotype in a slightly different way, as suggested by Ikeya and colleagues^45^ for *Drosophila*. Alternatively, and as pointed out for *Drosophila simulans*^46^, a short mating duration may be explained by a lower amount of transferred sperm when infected with *Wolbachia*.

Pre-mating sexual isolation between Peruvian and Brazilian-1 morphotypes has been previously demonstrated^30,35,36^. Because these morphotypes are infected by distinct *Wolbachia* strains, it was hypothesized that *Wolbachia* could, at least indirectly, be involved in this mechanism and therefore its removal would, partially or totally, revert the observed reproductive isolation. Here, the percentage of mated AfP females and AfC males was not affected by the infection status of the flies. However, when comparing the reciprocal crosses, a significant reduction in the percentage of mating was found when *Wolbachia* was removed from both sexes, which may have resulted from a detrimental effect of the antibiotic treatment on the mating competitiveness^47^. Thus, our results do not support the idea that *Wolbachia* infection could be one of the evolutionary forces behind the pre-zygotic isolation between the Peruvian and Brazilian-1 morphotypes. Regarding the time variables, even when statistical differences were found in some cases, they seem to be related to the morphotype *per se* more than to the infection status. These results are in concordance with previous results on mating latency and duration^30,35,36^.

As previously commented, the effects of antibiotic treatment on host physiology should be taken into account as a possible explanation for the effects on some biological parameters in crosses involving *Wolbachia*-cured individuals^48,49^. Despite the fact that the cured flies were reared for at least two generations without antibiotics before performing the experiments (allowing the reposition of the main gut microbiota), it is possible that some important symbionts have not been restored. If they played a role in mating propensity as suggested by Juárez *et al*.^47^, the curing process (alone or interacting with other factors) might have inadvertently and negatively affected the fitness of cured flies. Another factor that may have caused the observed differences among the experiments is the different genetic background of the two morphotypes. There are examples in which the same strain of *Wolbachia* has different effects in closely related hosts^39^. Transinfection experiments (i.e., *w*Per in cured AfC flies and *w*AfraCast2_A in cured AfP flies) may help to test this hypothesis.

Most known examples of reproductive phenotypes caused by *Wolbachia* are related to phenomena taking place after fertilization (i.e., post-zygotic effects). In our work, the infection status did not affect the egg-laying ability in any of the two morphotypes tested independently, and only a detrimental effect in the heterotypic cross between uninfected AfP females and AfC males was found. This reduction cannot be explained either by the origin of the flies or by their infection status and may be related to a specific biochemical stress associated to that particular combination of factors including the removal of undetected microorganisms potentially important to the normal development of the offspring. After egg-laying, uni-directional CI was confirmed for both morphotypes given the observed reduction in egg hatch, with an even higher intensity in the Peruvian morphotype. For AfC, there seems to be no effect after the larvae had hatched, which is evidenced in the similarity in the percentages of pupation and F1 emergence among crosses. Yet, AfP showed a significant decrease of pupation percentage only when both sexes were cured (and slightly decreased when only the female was cured) and this could be explained by some missing factors that co-evolved between host and bacteria (i.e., biochemical imbalance).

Cáceres *et al*.^30^ observed a reduction in egg hatch and a sex ratio distortion as a degree of post-zygotic isolation between flies from the Brazilian-1 and Peruvian morphotypes of *A. fraterculus*. These authors provided evidence suggesting major genetic differences based on the chromosomal asynapsis recorded in their hybrids. They also suggested the presence of a nuclear–cytoplasmic interaction, probably involved in the differences observed in reproductive parameters. Based on molecular, behavioural, and morphometric traits, the two morphotypes have accumulated important differences^30,34^, to the point in which they are considered undergoing speciation. In our study, no bi-directional CI was evidenced even though the two morphotypes are infected with two distinct *Wolbachia* strains. However, it is not known if these two strains, *w*Per and *w*AfraCast2_A, carry the same or closely related bacteriophage WO genes, *cifA* and *cifB*, that have recently been shown to contribute to the induction of -and in the case of *cifA* also in the rescue of- cytoplasmic incompatibility^50–53^. The fact that the cross of infected AfP female and infected AfC male has shown an even higher egg hatch percentage when compared with the respective control (i.e., the cured cross) might indicate that some other factors are potentially involved in these crosses that were not evaluated in our experiments. Further studies on mating compatibility between morphotypes considering their microbiota composition, interactions, and other physiological changes induced in the host species, will complement the information generated here to understand the host-symbionts dynamics and its association with phenotypic traits elicited.

In sum, we have evaluated the effects of the most widespread reproductive symbiont of insects infecting two morphotypes of the *A. fraterculus* cryptic species complex that show an intermediate degree of sexual isolation compared to other combinations of entities. Uni-directional CI was confirmed for both, with a different degree of penetrance. Bi-directional CI was not evidenced. Differences in the observed general pattern suggest biochemical imbalances produced directly or indirectly by the antibiotic treatment or by the absence of other relevant bacteria. Some other aspects, such as the characteristic density of *Wolbachia* of each morphotype, the impact of the antibiotic treatment on other symbionts, and transinfections, need further research to clarify the intricate evolution of *A. fraterculus*. Finally, our results contribute significant information about the role of this symbiont in life history traits such as mating behaviour and reproduction which are critical for the development and implementation of SIT and other related population suppression strategies against members of the *A. fraterculus* species complex.

## Material and Methods

### Insects

Peruvian and Brazilian-1 morphotypes of *A. fraterculus*^54^ were used as experimental models. The colony of the Peruvian morphotype was originally established in 2002 at the mass-rearing facility in La Molina, Lima, Peru from infested cherimoyas (*Annona cherimola* Miller). For the purpose of this paper we named it ‘AfP’. Flies from this stock were used to obtain the AfP infected and AfP cured colonies for the following experiments. The main stock of the Brazilian-1 morphotype derived from the experimental rearing kept at IGEAF (INTA Castelar, Argentina), which was named as ‘AfC’ and used as AfC infected colony. These colonies were established at the Insect Pest Control Laboratory (IPCL), FAO/IAEA, Seibersdorf, Austria, and reared according to Vera *et al*.^55^. A purified colony containing only *w*AfraCast2_A was obtained at IGEAF and sent to IPCL, which was treated with antibiotics (see below) and used as the AfC cured colony in the experiments. The AfC infected colony was previously established at IPCL from the same origin but contained both *Wolbachia* variants. It was considered that AfC cured and infected strains possess the same genetic background.

### Molecular detection and characterization of *Wolbachia*

Total DNA was isolated from 20 randomly selected adult flies (whole body) from each colony using the CTAB method^55^. *Wolbachia* detection was based on PCR and direct visualization of the present/absence of amplicons. For this purpose, a fragment (438 bp) of the gene encoding the 16S ribosomal RNA (*16S rRNA*) was amplified using the *Wolbachia*-specific primers wSpecF and wSpecR^56^. The results of PCR amplifications were visualized by electrophoresis in 1.5% w/v agarose gels and stained with ethidium bromide^55^. Images were captured with an UVP reveller (Fotodyne Inc. Hartland, WI, USA).

The identification of the *Wolbachia* strain infecting AfC and AfP colonies was performed by PCR amplification and sequence analysis of a portion (590 to 632 bp) of the *Wolbachia* surface protein (*wsp*) gene, obtained by PCR using 81F/691R primers^57^. Amplicons were purified using a Wizard SV Gel and PCR Clean-Up System (Promega) and sequenced. Forward and reverse sequences were obtained using an Abi 3130XL Genetic Analyzer (Applied Biosystem, Thermo Fisher Scientific Inc.). Sequences were manually edited and aligned using Bioedit^58^ and Staden Package^59^. The characterization of the sequences includes allele assignment based on comparisons of the nucleotide sequence obtained against the *Wolbachia* database and the analysis of hypervariable regions (HVRs), which were in turn determined by comparisons among available translated nucleotide sequences^60^. HVR allele definition is based on the analysis of the amino acid motifs of the *wsp* gene sequence (61–573 bp) in respect to *wMel*. Also, the sequences of five genes (*gat*B, *cox*A, *hcp*A, *fbp*A and *fts*Z) were analysed by means of an MLST-based approach as a further genetic characterization^61^. *Wolbachia* MLST public database was used for analyses and assignment of *wsp* alleles and MLST sequence type (https://pubmlst.org/wolbachia/).

### Antibiotic treatment

In order to obtain *Wolbachia*-cured individuals from the AfP colony, an antibiotic treatment was applied to the larval diet using 0.1% tetracycline (Sigma) at IPCL. Similarly, cured AfC individuals were obtained at IGEAF by application of 0.01% rifampicin (Laboratorios Richet, Buenos Aires, Argentina) in the larval diet. After 6 generations under antibiotic treatment, each colony was reared without antibiotic for at least two generations before the initiation of any experimental work. The cured status was confirmed through the 16S *rRNA* gene PCR approach as described above. In addition, the presence of other commonly present symbionts was tested using primers and conditions described previously (*Spiroplasma* sp.^62^, *Cardinium* sp.^63^, *Rickettsia* sp.^64^, and *Arsenophonus* sp.^65^).

The following colonies were considered for three experiments: Peruvian infected *A. fraterculus* (AfP+), Peruvian cured *A. fraterculus* (AfP−), Brazilian-1 infected *A. fraterculus* (AfC+), and Brazilian-1 cured *A. fraterculus* (AfC−). Each colony was reared in different cages following standard procedures^66^ and kept under environmentally controlled conditions (25 °C, 60% HR, 12 L:12D), with adult diet (sugar and hydrolysed yeast at a 3:1 ratio) and water.

### Incompatibility tests

AfP and AfC were first assessed independently in uni-directional cytoplasmic incompatibility (CI) tests, crossing individuals in four different ways (treatments) according to the sex and the infection status (Experiments 1 and 2 in ‘Results’ section). Also, infected or cured heterotypic crosses were performed (Experiment 3 in ‘Results’ section) in order to understand to what extent, the post-zygotic incompatibility reported between the two morphotypes^30^ is related to the infection by different *Wolbachia* strains (i.e. bi-directional CI).

#### Pre-zygotic incompatibility tests

In order to determine if the presence of *Wolbachia* affects mating compatibility, no-choice experiments were conducted with one virgin male and one virgin female inside a mating arena. To this end, adults were sorted by sex after emergence and kept in separate containers with adult food and water until sexual maturation (10 days after emergence for males^67^ and 14 days after emergence for females^68^). Around 8:30 h, virgin, 10–15 days-old males were singly introduced in acrylic boxes (10 × 10 × 13 cm) with a fine mesh on the sides and lid for proper ventilation, without food or water. Fifteen minutes later, one female was placed inside each box. For the experiment with AfP, 34, 50, 35, and 50 replicates for (AfP+ × AfP+), (AfP− × AfP−), (AfP+ × AfP−), and (AfP− × AfP+), respectively, were set up. For the experiment with AfC, 30, 45, 30, and 30 replicates for (AfC+ × AfC+), (AfC− × AfC−), (AfC+ × AfC−), and (AfC− × AfC+), respectively, were set up. For the experiment with AfP × AfC, 64, 50, 100, and 110 replicates for (AfP+ × AfC+), (AfC+ × AfP+), (AfP− × AfC−), and (AfC− × AfP−), respectively, were set up. As bi-directional CI can be revealed only when different and incompatible *Wolbachia* strains are present, no crosses involving both cured and infected flies were tested. Notation throughout the paper is female × male. The couples were checked continuously for mating occurrence. For each couple, the time of mating start and end were recorded. The experiment ended after a 30-minutes span in which no matings were recorded. Mated couples were then provided food and water and kept for further post-zygotic incompatibility tests.

#### Post-zygotic incompatibility tests

On the day after the mating test, mated females were offered an oviposition substrate. This device consisted of a Petri dish whose base (6 cm in diameter) was replaced by a black silicon-coated mesh. The dish was filled with water and placed on top of the acrylic box to allow constant oviposition through the mesh. Every two days, eggs from each female were transferred to a black filter paper and placed on larval diet within a small, plastic Petri dish (5 cm in diameter). Eggs were counted under a stereo-microscope (60 ×, Olympus, Japan). Samples with less than 10 eggs were discarded. Egg collection stopped after reaching at least 50 eggs per couple in more than one collection. Five to seven days after egg collection, hatched eggs were counted, the filter paper was removed, and the developing larvae were transferred into a closed, ventilated box with sawdust to allow pupation. In the case there were no hatched eggs, female spermathecae were dissected to check for sperm transfer following the protocol described by Segura *et al*.^69^. The females that showed empty spermathecae were not considered in the post-zygotic analyses. After all larvae had pupated, the Petri dish with the remaining larval diet was removed from the box to avoid fungal contamination. After emergence, the adults were counted, and their sex recorded. Emerged and non-emerged pupae were recorded as well. Parental couples were preserved in 96% ethanol to confirm the corresponding *Wolbachia* infection status (see procedures in section ‘Molecular detection and characterization of *Wolbachia*’).

All steps were carried out under controlled conditions of temperature (25 ± 2 °C), humidity (60 ± 10%) and photoperiod (14 L:10D).

After mating experiments, a total of 200 flies, representing all the combinations of morphotypes, sexes and infection status were screened for *Wolbachia* by the PCR-based protocol described above (16S *rRNA* gene amplification). Whenever one or both insects of the pair did not show the initially assigned status, the mating pair was eliminated from the data set and no longer considered in the statistical analyses of pre- and post-zygotic analyses.

### Statistical analyses

#### Pre-zygotic incompatibility tests

The percentage of matings was compared among crosses within each experiment using the number of mated and unmated couples in a Chi-square test of homogeneity. The latency to mate was calculated as the difference between the mating start time and the time at which females were released in the boxes. To standardize this value among treatments within each experiment, the lowest latency value obtained from each treatment was subtracted from the respective replicates. Latency was compared among crosses within each experiment by means of a one-way ANOVA. When assumptions were not met (Experiment 2), latency was transformed using log (latency + 1). The duration of the mating was calculated as the time elapsed between the starting and ending times of mating. This variable was analysed by means of a one-way ANOVA, using log(e) as transformation (Experiment 1 and 3) in order to meet homoscedasticity assumption.

#### Post-zygotic incompatibility tests

The percentage of females that laid at least 50 eggs (in more than one oviposition event) was compared among treatments by means of Chi-square test of homogeneity using the number of egg-laying females and non-egg-laying females. The percentage of egg hatch was calculated out of the total number of eggs collected in several opportunities for every mated female. This variable was analysed by means of a one-way ANOVA, using square root as transformation in those cases were the assumptions were not met (Experiment 1 and 2). The percentage of pupation was calculated as the percent of the hatched eggs that reached the pupal stage. A one-way ANOVA was used for testing differences among treatments. The percentage of adult emergence was compared among crosses by means of one-way ANOVA using rank transformation. Finally, sex ratio was calculated as the proportion of emerged females from the total number of emerged adults and it was compared among crosses by a one-way ANOVA. For Experiment 1, sex ratio was transformed to 1/(sex ratio +1) whereas data from Experiment 2 were log-transformed. For statistical purposes, cases with less than 10 eggs, pupae, or adults were not considered in the data analysis.

Whenever statistical differences in the Chi-square test of homogeneity were found, crosses were compared in pairs in order to detect the treatment responsible of the statistical significance. Significant p-values after ANOVAs were followed by Tukey tests. All statistical analyses were performed with STATISTICA version 7^70^.

## Supplementary information

Dataset 1

## Data Availability

All data generated or analysed during this study are included in this published article (and its Supplementary Information file). *Wsp* gene sequences generated in this study from *w*AfraCast2_A have been deposited in GenBank, National Center for Biotechnology Information (NCBI) databases under accession number KC589027.1. Allelic profile of MLST scheme of five genes (*gatB, coxA, hcpA, fbpA and ftsZ*) and HVRs allelic profile of *w*AfraCast2_A are available on *Wolbachia* MLST database. Nucleotide sequences of the *groEL, gltA, dnaA, sucB, aspC, atpD* and *pdhB* genes from *Wolbachia* infecting Brazilian-1 A. *fraterculus* were submitted to GenBank under accession numbers MG977022-28 respectively. Nucleotide sequences of MLST from *Wolbachia* infecting Peruvian A. fraterculus corresponding to *gatB, coxA, hcpA, fbpA, ftsZ* and *hcpA* genes were deposited in GenBank under AN MN145458-MN145462”.
