# Supplementary material for: Symbionts do not affect the mating incompatibility between the Brazilian-1 and Peruvian morphotypes of the Anastrepha fraterculus cryptic species complex
Source: Sci Rep. 2019 Dec 4;9:18319. doi: 10.1038/s41598-019-54704-y (PMC6893037; doi:10.1038/s41598-019-54704-y)
Supplement: Supplementary file 1 — Dataset 1 [file 41598_2019_54704_MOESM1_ESM.docx]

**Symbionts do not affect the mating incompatibility between the Brazilian-1 and Peruvian morphotypes of the *Anastrepha fraterculus* cryptic species complex**

Francisco Devescovi^1,2,+^, Claudia A. Conte^2,+^, Antonis A. Augustinos^,3$^, Elena I. Cancio Martinez^3^, Diego F. Segura^1,2^, Carlos E. Cáceres^3^, Silvia B. Lanzavecchia^2^, Kostas Bourtzis^3*^

^*^K.Bourtzis@iaea.org

^+^These authors contributed equally to this work

^1^Consejo Nacional de Investigaciones Científicas y Técnicas, Ciudad Autónoma de Buenos Aires, C1425FQB, Argentina.

^2^Instituto de Genética “E.A. Favret”, Instituto Nacional de Tecnología Agropecuaria, Hurlingham, B1686, Buenos Aires, Argentina.

^3^Insect Pest Control Laboratory, Joint FAO/IAEA Division of Nuclear Techniques in Food and Agriculture, Vienna International Centre, P.O. Box 100, 1400, Vienna, Austria.

^$^Present address: Department of Plant Protection, Institute of Industrial and Forage Crops, Hellenic Agricultural Organization – DEMETER, Patras, Greece.

| Colony | Treatment | Latency to mate (min) |  |  |  |  |
| --- | --- | --- | --- | --- | --- | --- |
| Peru | ♀AfP(+) x ♂AfP(+) | 163 |  |  |  |  |
|  |  | 176 |  |  |  |  |
|  |  | 177 |  |  |  |  |
|  |  | 100 |  |  |  |  |
|  |  | 116 |  |  |  |  |
|  |  | 148 |  |  |  |  |
|  |  | 185 |  |  |  |  |
|  |  | 130 |  |  |  |  |
|  |  | 204 |  |  |  |  |
|  |  | 83 |  |  |  |  |
|  |  | 178 |  |  |  |  |
|  |  | 163 |  |  |  |  |
|  |  | 95 |  |  |  |  |
|  |  | 177 |  |  |  |  |
|  |  | 50 |  |  |  |  |
|  |  | 157 |  |  |  |  |
|  |  | 147 |  |  |  |  |
|  |  | 195 |  |  |  |  |
|  |  | 173 |  |  |  |  |
|  |  | 158 |  |  |  |  |
|  |  | 200 |  |  |  |  |
|  |  | 155 |  |  |  |  |
|  |  | 98 |  |  |  |  |
|  |  | 22 |  |  |  |  |
|  |  | 40 |  |  |  |  |
|  | ♀AfP(-) x ♂AfP(-) | 110 |  |  |  |  |
|  |  | 14 |  |  |  |  |
|  |  | 20 |  |  |  |  |
|  |  | 168 |  |  |  |  |
|  |  | 48 |  |  |  |  |
|  |  | 20 |  |  |  |  |
|  |  | 10 |  |  |  |  |
|  |  | 80 |  |  |  |  |
|  |  | 21 |  |  |  |  |
|  |  | 166 |  |  |  |  |
|  |  | 62 |  |  |  |  |
|  |  | 102 |  |  |  |  |
|  |  | 136 |  |  |  |  |
|  |  | 48 |  |  |  |  |
|  |  | 38 |  |  |  |  |
|  |  | 97 |  |  |  |  |
|  |  | 155 |  |  |  |  |
|  |  | 42 |  |  |  |  |
|  |  | 30 |  |  |  |  |
|  |  | 30 |  |  |  |  |
|  |  | 30 |  |  |  |  |
|  |  | 87 |  |  |  |  |
|  |  | 17 |  |  |  |  |
|  |  | 51 |  |  |  |  |
|  |  | 87 |  |  |  |  |
|  |  | 143 |  |  |  |  |
|  |  | 8 |  |  |  |  |
|  |  | 152 |  |  |  |  |
|  |  | 232 |  |  |  |  |
|  |  | 112 |  |  |  |  |
|  |  | 2 |  |  |  |  |
|  | ♀AfP(+) x ♂AfP(-) | 36 |  |  |  |  |
|  |  | 86 |  |  |  |  |
|  |  | 249 |  |  |  |  |
|  |  | 128 |  |  |  |  |
|  |  | 49 |  |  |  |  |
|  |  | 131 |  |  |  |  |
|  |  | 257 |  |  |  |  |
|  |  | 159 |  |  |  |  |
|  |  | 231 |  |  |  |  |
|  |  | 73 |  |  |  |  |
|  |  | 185 |  |  |  |  |
|  |  | 129 |  |  |  |  |
|  |  | 168 |  |  |  |  |
|  |  | 51 |  |  |  |  |
|  |  | 65 |  |  |  |  |
|  |  | 91 |  |  |  |  |
|  |  | 118 |  |  |  |  |
|  |  | 255 |  |  |  |  |
|  |  | 127 |  |  |  |  |
|  |  | 142 |  |  |  |  |
|  |  | 89 |  |  |  |  |
|  |  | 259 |  |  |  |  |
|  |  | 250 |  |  |  |  |
|  |  | 217 |  |  |  |  |
|  |  | 190 |  |  |  |  |
|  | ♀AfP(-) x ♂AfP(+) | 76 |  |  |  |  |
|  |  | 2 |  |  |  |  |
|  |  | 231 |  |  |  |  |
|  |  | 123 |  |  |  |  |
|  |  | 118 |  |  |  |  |
|  |  | 112 |  |  |  |  |
|  |  | 103 |  |  |  |  |
|  |  | 89 |  |  |  |  |
|  |  | 137 |  |  |  |  |
|  |  | 98 |  |  |  |  |
|  |  | 109 |  |  |  |  |
|  |  | 73 |  |  |  |  |
|  |  | 123 |  |  |  |  |
|  |  | 89 |  |  |  |  |
|  |  | 137 |  |  |  |  |
|  |  | 115 |  |  |  |  |
|  |  | 148 |  |  |  |  |
|  |  | 134 |  |  |  |  |
|  |  | 223 |  |  |  |  |
|  |  | 137 |  |  |  |  |
|  |  | 221 |  |  |  |  |
|  |  | 41 |  |  |  |  |
|  |  | 260 |  |  |  |  |
|  |  | 177 |  |  |  |  |
|  |  | 110 |  |  |  |  |
|  |  | 95 |  |  |  |  |
|  |  | 40 |  |  |  |  |
|  |  | 210 |  |  |  |  |
|  |  | 125 |  |  |  |  |
|  |  | 125 |  |  |  |  |
|  |  | 135 |  |  |  |  |
|  |  | 215 |  |  |  |  |
|  |  | 110 |  |  |  |  |
|  |  | 125 |  |  |  |  |
| Castelar | ♀AfC(+) x ♂AfC(+) | 3 |  |  |  |  |
|  |  | 3 |  |  |  |  |
|  |  | 3 |  |  |  |  |
|  |  | 3 |  |  |  |  |
|  |  | 3 |  |  |  |  |
|  |  | 3 |  |  |  |  |
|  |  | 3 |  |  |  |  |
|  |  | 3 |  |  |  |  |
|  |  | 3 |  |  |  |  |
|  |  | 3 |  |  |  |  |
|  |  | 3 |  |  |  |  |
|  |  | 3 |  |  |  |  |
|  |  | 3 |  |  |  |  |
|  |  | 3 |  |  |  |  |
|  |  | 3 |  |  |  |  |
|  |  | 3 |  |  |  |  |
|  |  | 3 |  |  |  |  |
|  |  | 3 |  |  |  |  |
|  |  | 3 |  |  |  |  |
|  |  | 3 |  |  |  |  |
|  |  | 3 |  |  |  |  |
|  |  | 3 |  |  |  |  |
|  |  | 3 |  |  |  |  |
|  |  | 3 |  |  |  |  |
|  |  | 70 |  |  |  |  |
|  | ♀AfC(-) x ♂AfC(-) | 14 |  |  |  |  |
|  |  | 14 |  |  |  |  |
|  |  | 14 |  |  |  |  |
|  |  | 16 |  |  |  |  |
|  |  | 16 |  |  |  |  |
|  |  | 1 |  |  |  |  |
|  |  | 1 |  |  |  |  |
|  |  | 1 |  |  |  |  |
|  |  | 1 |  |  |  |  |
|  |  | 1 |  |  |  |  |
|  |  | 1 |  |  |  |  |
|  |  | 1 |  |  |  |  |
|  |  | 1 |  |  |  |  |
|  |  | 1 |  |  |  |  |
|  |  | 1 |  |  |  |  |
|  |  | 1 |  |  |  |  |
|  |  | 1 |  |  |  |  |
|  |  | 1 |  |  |  |  |
|  |  | 1 |  |  |  |  |
|  |  | 18 |  |  |  |  |
|  |  | 25 |  |  |  |  |
|  |  | 36 |  |  |  |  |
|  |  | 68 |  |  |  |  |
|  | ♀AfC(+) x ♂AfC(-) | 59 |  |  |  |  |
|  |  | 4 |  |  |  |  |
|  |  | 4 |  |  |  |  |
|  |  | 4 |  |  |  |  |
|  |  | 4 |  |  |  |  |
|  |  | 4 |  |  |  |  |
|  |  | 81 |  |  |  |  |
|  |  | 4 |  |  |  |  |
|  |  | 4 |  |  |  |  |
|  |  | 4 |  |  |  |  |
|  |  | 4 |  |  |  |  |
|  |  | 4 |  |  |  |  |
|  |  | 4 |  |  |  |  |
|  |  | 4 |  |  |  |  |
|  |  | 4 |  |  |  |  |
|  |  | 4 |  |  |  |  |
|  |  | 4 |  |  |  |  |
|  |  | 4 |  |  |  |  |
|  |  | 4 |  |  |  |  |
|  |  | 4 |  |  |  |  |
|  |  | 4 |  |  |  |  |
|  | ♀AfC(-) x ♂AfC(+) | 3 |  |  |  |  |
|  |  | 44 |  |  |  |  |
|  |  | 3 |  |  |  |  |
|  |  | 3 |  |  |  |  |
|  |  | 3 |  |  |  |  |
|  |  | 3 |  |  |  |  |
|  |  | 3 |  |  |  |  |
|  |  | 3 |  |  |  |  |
|  |  | 3 |  |  |  |  |
|  |  | 3 |  |  |  |  |
|  |  | 3 |  |  |  |  |
|  |  | 3 |  |  |  |  |
|  |  | 3 |  |  |  |  |
|  |  | 3 |  |  |  |  |
|  |  | 3 |  |  |  |  |
|  |  | 3 |  |  |  |  |
|  |  | 3 |  |  |  |  |
|  |  | 3 |  |  |  |  |
| Peru x Castelar | ♀AfP(+) x ♂AfC(+) | 335 |  |  |  |  |
|  |  | 125 |  |  |  |  |
|  |  | 20 |  |  |  |  |
|  |  | 283 |  |  |  |  |
|  |  | 240 |  |  |  |  |
|  |  | 190 |  |  |  |  |
|  |  | 45 |  |  |  |  |
|  |  | 10 |  |  |  |  |
|  |  | 61 |  |  |  |  |
|  |  | 145 |  |  |  |  |
|  |  | 27 |  |  |  |  |
|  |  | 45 |  |  |  |  |
|  |  | 176 |  |  |  |  |
|  |  | 223 |  |  |  |  |
|  |  | 13 |  |  |  |  |
|  |  | 29 |  |  |  |  |
|  |  | 133 |  |  |  |  |
|  |  | 21 |  |  |  |  |
|  |  | 13 |  |  |  |  |
|  |  | 123 |  |  |  |  |
|  |  | 128 |  |  |  |  |
|  |  | 13 |  |  |  |  |
|  |  | 133 |  |  |  |  |
|  |  | 13 |  |  |  |  |
|  | ♀AfC(+) x ♂AfP(+) | 319 |  |  |  |  |
|  |  | 229 |  |  |  |  |
|  |  | 284 |  |  |  |  |
|  |  | 9 |  |  |  |  |
|  |  | 114 |  |  |  |  |
|  |  | 165 |  |  |  |  |
|  |  | 104 |  |  |  |  |
|  |  | 179 |  |  |  |  |
|  |  | 301 |  |  |  |  |
|  |  | 114 |  |  |  |  |
|  |  | 76 |  |  |  |  |
|  |  | 165 |  |  |  |  |
|  |  | 194 |  |  |  |  |
|  |  | 9 |  |  |  |  |
|  |  | 9 |  |  |  |  |
|  |  | 229 |  |  |  |  |
|  |  | 279 |  |  |  |  |
|  |  | 24 |  |  |  |  |
|  |  | 204 |  |  |  |  |
|  |  | 264 |  |  |  |  |
|  |  | 301 |  |  |  |  |
|  |  | 8 |  |  |  |  |
|  |  | 8 |  |  |  |  |
|  |  | 68 |  |  |  |  |
|  |  | 8 |  |  |  |  |
|  |  | 24 |  |  |  |  |
|  |  | 16 |  |  |  |  |
|  |  | 24 |  |  |  |  |
|  |  | 8 |  |  |  |  |
|  | ♀AfP(-) x ♂AfC(-) | 325 |  |  |  |  |
|  |  | 380 |  |  |  |  |
|  |  | 145 |  |  |  |  |
|  |  | 366 |  |  |  |  |
|  |  | 277 |  |  |  |  |
|  |  | 182 |  |  |  |  |
|  |  | 435 |  |  |  |  |
|  |  | 7 |  |  |  |  |
|  |  | 276 |  |  |  |  |
|  |  | 250 |  |  |  |  |
|  |  | 185 |  |  |  |  |
|  |  | 70 |  |  |  |  |
|  |  | 225 |  |  |  |  |
|  |  | 370 |  |  |  |  |
|  |  | 250 |  |  |  |  |
|  |  | 20 |  |  |  |  |
|  |  | 80 |  |  |  |  |
|  |  | 52 |  |  |  |  |
|  |  | 275 |  |  |  |  |
|  |  | 115 |  |  |  |  |
|  |  | 46 |  |  |  |  |
|  |  | 300 |  |  |  |  |
|  |  | 90 |  |  |  |  |
|  |  | 258 |  |  |  |  |
|  |  | 160 |  |  |  |  |
|  |  | 5 |  |  |  |  |
|  |  | 160 |  |  |  |  |
|  |  | 270 |  |  |  |  |
|  |  | 160 |  |  |  |  |
|  |  | 20 |  |  |  |  |
|  |  | 160 |  |  |  |  |
|  |  | 160 |  |  |  |  |
|  |  | 160 |  |  |  |  |
|  |  | 145 |  |  |  |  |
|  |  | 20 |  |  |  |  |
|  |  | 20 |  |  |  |  |
|  |  | 115 |  |  |  |  |
|  |  | 80 |  |  |  |  |
|  |  | 20 |  |  |  |  |
|  |  | 20 |  |  |  |  |
|  |  | 250 |  |  |  |  |
|  | ♀AfC(-) x ♂AfP(-) | 30 |  |  |  |  |
|  |  | 70 |  |  |  |  |
|  |  | 22 |  |  |  |  |
|  |  | 30 |  |  |  |  |
|  |  | 135 |  |  |  |  |
|  |  | 15 |  |  |  |  |
|  |  | 45 |  |  |  |  |
|  |  | 177 |  |  |  |  |
|  |  | 155 |  |  |  |  |
|  |  | 41 |  |  |  |  |
|  |  | 183 |  |  |  |  |
|  |  | 20 |  |  |  |  |
|  |  | 33 |  |  |  |  |
|  |  | 65 |  |  |  |  |
|  |  | 173 |  |  |  |  |
|  |  | 93 |  |  |  |  |
|  |  | 173 |  |  |  |  |
|  |  | 188 |  |  |  |  |
|  |  | 59 |  |  |  |  |
|  |  | 213 |  |  |  |  |
|  |  | 20 |  |  |  |  |
|  |  | 211 |  |  |  |  |
|  |  | 26 |  |  |  |  |
|  |  | 83 |  |  |  |  |
|  |  | 33 |  |  |  |  |
|  |  | 133 |  |  |  |  |
|  |  | 263 |  |  |  |  |
|  |  | 223 |  |  |  |  |
|  |  | 41 |  |  |  |  |
|  |  | 223 |  |  |  |  |
|  |  | 33 |  |  |  |  |
|  |  |  |  |  |  |  |
|  |  |  |  |  |  |  |
| Colony | Treatment | Mating duration (min) |  |  |  |  |
| Peru | ♀AfP(+) x ♂AfP(+) | 9 |  |  |  |  |
|  |  | 9 |  |  |  |  |
|  |  | 12 |  |  |  |  |
|  |  | 13 |  |  |  |  |
|  |  | 13 |  |  |  |  |
|  |  | 14 |  |  |  |  |
|  |  | 14 |  |  |  |  |
|  |  | 16 |  |  |  |  |
|  |  | 18 |  |  |  |  |
|  |  | 18 |  |  |  |  |
|  |  | 19 |  |  |  |  |
|  |  | 20 |  |  |  |  |
|  |  | 20 |  |  |  |  |
|  |  | 21 |  |  |  |  |
|  |  | 21 |  |  |  |  |
|  |  | 22 |  |  |  |  |
|  |  | 27 |  |  |  |  |
|  |  | 30 |  |  |  |  |
|  |  | 30 |  |  |  |  |
|  |  | 32 |  |  |  |  |
|  |  | 34 |  |  |  |  |
|  |  | 42 |  |  |  |  |
|  |  | 42 |  |  |  |  |
|  |  | 61 |  |  |  |  |
|  |  | 80 |  |  |  |  |
|  | ♀AfP(-) x ♂AfP(-) | 18 |  |  |  |  |
|  |  | 21 |  |  |  |  |
|  |  | 22 |  |  |  |  |
|  |  | 23 |  |  |  |  |
|  |  | 24 |  |  |  |  |
|  |  | 25 |  |  |  |  |
|  |  | 28 |  |  |  |  |
|  |  | 32 |  |  |  |  |
|  |  | 33 |  |  |  |  |
|  |  | 34 |  |  |  |  |
|  |  | 34 |  |  |  |  |
|  |  | 37 |  |  |  |  |
|  |  | 39 |  |  |  |  |
|  |  | 40 |  |  |  |  |
|  |  | 40 |  |  |  |  |
|  |  | 40 |  |  |  |  |
|  |  | 45 |  |  |  |  |
|  |  | 46 |  |  |  |  |
|  |  | 47 |  |  |  |  |
|  |  | 48 |  |  |  |  |
|  |  | 50 |  |  |  |  |
|  |  | 52 |  |  |  |  |
|  |  | 57 |  |  |  |  |
|  |  | 57 |  |  |  |  |
|  |  | 57 |  |  |  |  |
|  |  | 60 |  |  |  |  |
|  |  | 70 |  |  |  |  |
|  |  | 70 |  |  |  |  |
|  |  | 80 |  |  |  |  |
|  |  | 81 |  |  |  |  |
|  |  | 86 |  |  |  |  |
|  |  | 89 |  |  |  |  |
|  |  | 95 |  |  |  |  |
|  |  | 108 |  |  |  |  |
|  |  | 116 |  |  |  |  |
|  |  | 123 |  |  |  |  |
|  |  | 132 |  |  |  |  |
|  |  | 140 |  |  |  |  |
|  |  | 189 |  |  |  |  |
|  |  | 193 |  |  |  |  |
|  | ♀AfP(+) x ♂AfP(-) | 7 |  |  |  |  |
|  |  | 10 |  |  |  |  |
|  |  | 16 |  |  |  |  |
|  |  | 17 |  |  |  |  |
|  |  | 18 |  |  |  |  |
|  |  | 19 |  |  |  |  |
|  |  | 20 |  |  |  |  |
|  |  | 22 |  |  |  |  |
|  |  | 23 |  |  |  |  |
|  |  | 24 |  |  |  |  |
|  |  | 26 |  |  |  |  |
|  |  | 27 |  |  |  |  |
|  |  | 28 |  |  |  |  |
|  |  | 29 |  |  |  |  |
|  |  | 29 |  |  |  |  |
|  |  | 29 |  |  |  |  |
|  |  | 30 |  |  |  |  |
|  |  | 31 |  |  |  |  |
|  |  | 31 |  |  |  |  |
|  |  | 33 |  |  |  |  |
|  |  | 44 |  |  |  |  |
|  |  | 48 |  |  |  |  |
|  |  | 50 |  |  |  |  |
|  |  | 53 |  |  |  |  |
|  |  | 168 |  |  |  |  |
|  | ♀AfP(-) x ♂AfP(+) | 13 |  |  |  |  |
|  |  | 22 |  |  |  |  |
|  |  | 32 |  |  |  |  |
|  |  | 33 |  |  |  |  |
|  |  | 35 |  |  |  |  |
|  |  | 36 |  |  |  |  |
|  |  | 38 |  |  |  |  |
|  |  | 40 |  |  |  |  |
|  |  | 41 |  |  |  |  |
|  |  | 41 |  |  |  |  |
|  |  | 44 |  |  |  |  |
|  |  | 45 |  |  |  |  |
|  |  | 48 |  |  |  |  |
|  |  | 50 |  |  |  |  |
|  |  | 50 |  |  |  |  |
|  |  | 54 |  |  |  |  |
|  |  | 58 |  |  |  |  |
|  |  | 61 |  |  |  |  |
|  |  | 62 |  |  |  |  |
|  |  | 65 |  |  |  |  |
|  |  | 65 |  |  |  |  |
|  |  | 66 |  |  |  |  |
|  |  | 82 |  |  |  |  |
|  |  | 83 |  |  |  |  |
|  |  | 85 |  |  |  |  |
|  |  | 88 |  |  |  |  |
|  |  | 93 |  |  |  |  |
|  |  | 101 |  |  |  |  |
|  |  | 108 |  |  |  |  |
|  |  | 156 |  |  |  |  |
|  |  | 171 |  |  |  |  |
| Castelar | ♀AfC(+) x ♂AfC(+) | 18 |  |  |  |  |
|  |  | 20 |  |  |  |  |
|  |  | 24 |  |  |  |  |
|  |  | 38 |  |  |  |  |
|  |  | 38 |  |  |  |  |
|  |  | 38 |  |  |  |  |
|  |  | 43 |  |  |  |  |
|  |  | 43 |  |  |  |  |
|  |  | 45 |  |  |  |  |
|  |  | 50 |  |  |  |  |
|  |  | 50 |  |  |  |  |
|  |  | 50 |  |  |  |  |
|  |  | 50 |  |  |  |  |
|  |  | 60 |  |  |  |  |
|  |  | 60 |  |  |  |  |
|  |  | 60 |  |  |  |  |
|  |  | 60 |  |  |  |  |
|  |  | 60 |  |  |  |  |
|  |  | 60 |  |  |  |  |
|  |  | 67 |  |  |  |  |
|  |  | 72 |  |  |  |  |
|  |  | 72 |  |  |  |  |
|  |  | 79 |  |  |  |  |
|  |  | 87 |  |  |  |  |
|  |  | 104 |  |  |  |  |
|  | ♀AfC(-) x ♂AfC(-) | 24 |  |  |  |  |
|  |  | 32 |  |  |  |  |
|  |  | 58 |  |  |  |  |
|  |  | 63 |  |  |  |  |
|  |  | 64 |  |  |  |  |
|  |  | 64 |  |  |  |  |
|  |  | 70 |  |  |  |  |
|  |  | 76 |  |  |  |  |
|  |  | 79 |  |  |  |  |
|  |  | 81 |  |  |  |  |
|  |  | 86 |  |  |  |  |
|  |  | 86 |  |  |  |  |
|  |  | 86 |  |  |  |  |
|  |  | 86 |  |  |  |  |
|  |  | 86 |  |  |  |  |
|  |  | 98 |  |  |  |  |
|  |  | 98 |  |  |  |  |
|  |  | 98 |  |  |  |  |
|  |  | 105 |  |  |  |  |
|  |  | 108 |  |  |  |  |
|  |  | 113 |  |  |  |  |
|  |  | 119 |  |  |  |  |
|  | ♀AfC(+) x ♂AfC(-) | 21 |  |  |  |  |
|  |  | 35 |  |  |  |  |
|  |  | 43 |  |  |  |  |
|  |  | 43 |  |  |  |  |
|  |  | 43 |  |  |  |  |
|  |  | 43 |  |  |  |  |
|  |  | 55 |  |  |  |  |
|  |  | 55 |  |  |  |  |
|  |  | 55 |  |  |  |  |
|  |  | 55 |  |  |  |  |
|  |  | 55 |  |  |  |  |
|  |  | 61 |  |  |  |  |
|  |  | 65 |  |  |  |  |
|  |  | 65 |  |  |  |  |
|  |  | 68 |  |  |  |  |
|  |  | 72 |  |  |  |  |
|  |  | 72 |  |  |  |  |
|  |  | 72 |  |  |  |  |
|  |  | 77 |  |  |  |  |
|  |  | 77 |  |  |  |  |
|  |  | 92 |  |  |  |  |
|  | ♀AfC(-) x ♂AfC(+) | 37 |  |  |  |  |
|  |  | 38 |  |  |  |  |
|  |  | 57 |  |  |  |  |
|  |  | 57 |  |  |  |  |
|  |  | 69 |  |  |  |  |
|  |  | 69 |  |  |  |  |
|  |  | 69 |  |  |  |  |
|  |  | 69 |  |  |  |  |
|  |  | 69 |  |  |  |  |
|  |  | 79 |  |  |  |  |
|  |  | 79 |  |  |  |  |
|  |  | 86 |  |  |  |  |
|  |  | 86 |  |  |  |  |
|  |  | 91 |  |  |  |  |
|  |  | 91 |  |  |  |  |
|  |  | 98 |  |  |  |  |
|  |  | 98 |  |  |  |  |
| Peru x Castelar | ♀AfP(+) x ♂AfC(+) | 10 |  |  |  |  |
|  |  | 11 |  |  |  |  |
|  |  | 11 |  |  |  |  |
|  |  | 12 |  |  |  |  |
|  |  | 12 |  |  |  |  |
|  |  | 12 |  |  |  |  |
|  |  | 14 |  |  |  |  |
|  |  | 14 |  |  |  |  |
|  |  | 16 |  |  |  |  |
|  |  | 18 |  |  |  |  |
|  |  | 18 |  |  |  |  |
|  |  | 19 |  |  |  |  |
|  |  | 20 |  |  |  |  |
|  |  | 20 |  |  |  |  |
|  |  | 20 |  |  |  |  |
|  |  | 20 |  |  |  |  |
|  |  | 22 |  |  |  |  |
|  |  | 25 |  |  |  |  |
|  |  | 25 |  |  |  |  |
|  |  | 25 |  |  |  |  |
|  |  | 32 |  |  |  |  |
|  |  | 40 |  |  |  |  |
|  |  | 64 |  |  |  |  |
|  | ♀AfC(+) x ♂AfP(+) | 7 |  |  |  |  |
|  |  | 20 |  |  |  |  |
|  |  | 25 |  |  |  |  |
|  |  | 26 |  |  |  |  |
|  |  | 27 |  |  |  |  |
|  |  | 31 |  |  |  |  |
|  |  | 32 |  |  |  |  |
|  |  | 32 |  |  |  |  |
|  |  | 34 |  |  |  |  |
|  |  | 34 |  |  |  |  |
|  |  | 35 |  |  |  |  |
|  |  | 40 |  |  |  |  |
|  |  | 40 |  |  |  |  |
|  |  | 40 |  |  |  |  |
|  |  | 40 |  |  |  |  |
|  |  | 42 |  |  |  |  |
|  |  | 43 |  |  |  |  |
|  |  | 46 |  |  |  |  |
|  |  | 48 |  |  |  |  |
|  |  | 50 |  |  |  |  |
|  |  | 50 |  |  |  |  |
|  |  | 55 |  |  |  |  |
|  |  | 59 |  |  |  |  |
|  |  | 60 |  |  |  |  |
|  |  | 60 |  |  |  |  |
|  |  | 61 |  |  |  |  |
|  |  | 65 |  |  |  |  |
|  |  | 75 |  |  |  |  |
|  |  | 81 |  |  |  |  |
|  | ♀AfP(-) x ♂AfC(-) | 8 |  |  |  |  |
|  |  | 10 |  |  |  |  |
|  |  | 12 |  |  |  |  |
|  |  | 12 |  |  |  |  |
|  |  | 14 |  |  |  |  |
|  |  | 15 |  |  |  |  |
|  |  | 15 |  |  |  |  |
|  |  | 15 |  |  |  |  |
|  |  | 15 |  |  |  |  |
|  |  | 15 |  |  |  |  |
|  |  | 18 |  |  |  |  |
|  |  | 20 |  |  |  |  |
|  |  | 20 |  |  |  |  |
|  |  | 20 |  |  |  |  |
|  |  | 20 |  |  |  |  |
|  |  | 20 |  |  |  |  |
|  |  | 22 |  |  |  |  |
|  |  | 25 |  |  |  |  |
|  |  | 25 |  |  |  |  |
|  |  | 25 |  |  |  |  |
|  |  | 25 |  |  |  |  |
|  |  | 25 |  |  |  |  |
|  |  | 27 |  |  |  |  |
|  |  | 28 |  |  |  |  |
|  |  | 30 |  |  |  |  |
|  |  | 30 |  |  |  |  |
|  |  | 30 |  |  |  |  |
|  |  | 30 |  |  |  |  |
|  |  | 30 |  |  |  |  |
|  |  | 30 |  |  |  |  |
|  |  | 30 |  |  |  |  |
|  |  | 30 |  |  |  |  |
|  |  | 32 |  |  |  |  |
|  |  | 34 |  |  |  |  |
|  |  | 36 |  |  |  |  |
|  |  | 36 |  |  |  |  |
|  |  | 38 |  |  |  |  |
|  |  | 45 |  |  |  |  |
|  |  | 54 |  |  |  |  |
|  |  | 60 |  |  |  |  |
|  | ♀AfC(-) x ♂AfP(-) | 20 |  |  |  |  |
|  |  | 20 |  |  |  |  |
|  |  | 20 |  |  |  |  |
|  |  | 22 |  |  |  |  |
|  |  | 27 |  |  |  |  |
|  |  | 27 |  |  |  |  |
|  |  | 29 |  |  |  |  |
|  |  | 35 |  |  |  |  |
|  |  | 35 |  |  |  |  |
|  |  | 39 |  |  |  |  |
|  |  | 40 |  |  |  |  |
|  |  | 42 |  |  |  |  |
|  |  | 43 |  |  |  |  |
|  |  | 47 |  |  |  |  |
|  |  | 48 |  |  |  |  |
|  |  | 48 |  |  |  |  |
|  |  | 49 |  |  |  |  |
|  |  | 50 |  |  |  |  |
|  |  | 52 |  |  |  |  |
|  |  | 53 |  |  |  |  |
|  |  | 60 |  |  |  |  |
|  |  | 62 |  |  |  |  |
|  |  | 63 |  |  |  |  |
|  |  | 65 |  |  |  |  |
|  |  | 65 |  |  |  |  |
|  |  | 70 |  |  |  |  |
|  |  | 70 |  |  |  |  |
|  |  | 80 |  |  |  |  |
|  |  | 105 |  |  |  |  |
|  |  | 125 |  |  |  |  |
|  |  |  |  |  |  |  |
|  |  |  |  |  |  |  |
| Colony | Treatment | total eggs | hatched eggs | Egg hatch (%) |  |  |
| Peru | ♀AfP(-) x ♂AfP(-) | 53 | 42 | 79,25 |  |  |
|  |  | 64 | 19 | 29,69 |  |  |
|  |  | 103 | 22 | 21,36 |  |  |
|  |  | 158 | 29 | 15,68 |  |  |
|  |  | 161 | 12 | 7,45 |  |  |
|  |  | 115 | 25 | 21,74 |  |  |
|  |  | 116 | 47 | 40,52 |  |  |
|  |  | 144 | 120 | 83,33 |  |  |
|  |  | 337 | 99 | 29,38 |  |  |
|  |  | 169 | 94 | 55,62 |  |  |
|  |  | 109 | 67 | 61,47 |  |  |
|  |  | 114 | 96 | 84,21 |  |  |
|  |  | 130 | 94 | 72,31 |  |  |
|  |  | 179 | 96 | 53,63 |  |  |
|  |  | 186 | 122 | 65,59 |  |  |
|  |  | 206 | 54 | 26,21 |  |  |
|  |  | 217 | 148 | 68,20 |  |  |
|  |  | 157 | 152 | 96,82 |  |  |
|  |  | 230 | 208 | 90,43 |  |  |
|  |  | 286 | 207 | 72,38 |  |  |
|  |  | 264 | 230 | 87,12 |  |  |
|  |  | 372 | 322 | 86,56 |  |  |
|  | ♀AfP(+) x ♂AfP(+) | 125 | 11 | 8,80 |  |  |
|  |  | 106 | 39 | 36,79 |  |  |
|  |  | 217 | 59 | 27,19 |  |  |
|  |  | 91 | 45 | 49,45 |  |  |
|  |  | 126 | 70 | 55,56 |  |  |
|  |  | 134 | 99 | 73,88 |  |  |
|  |  | 172 | 107 | 62,21 |  |  |
|  |  | 165 | 48 | 29,09 |  |  |
|  |  | 120 | 103 | 85,83 |  |  |
|  |  | 200 | 137 | 68,50 |  |  |
|  |  | 221 | 168 | 76,02 |  |  |
|  |  | 257 | 141 | 54,86 |  |  |
|  |  | 240 | 196 | 81,67 |  |  |
|  |  | 248 | 149 | 60,08 |  |  |
|  |  | 157 | 141 | 89,81 |  |  |
|  |  | 273 | 157 | 57,51 |  |  |
|  |  | 283 | 166 | 58,66 |  |  |
|  |  | 233 | 170 | 72,96 |  |  |
|  |  | 336 | 327 | 97,32 |  |  |
|  | ♀AfP(+) x ♂AfP(-) | 138 | 29 | 21,01 |  |  |
|  |  | 300 | 59 | 19,67 |  |  |
|  |  | 103 | 53 | 51,46 |  |  |
|  |  | 179 | 81 | 45,25 |  |  |
|  |  | 49 | 41 | 83,67 |  |  |
|  |  | 167 | 96 | 57,49 |  |  |
|  |  | 132 | 81 | 61,36 |  |  |
|  |  | 110 | 107 | 97,27 |  |  |
|  |  | 121 | 106 | 87,60 |  |  |
|  |  | 246 | 153 | 62,20 |  |  |
|  |  | 172 | 166 | 96,51 |  |  |
|  |  | 200 | 194 | 97,00 |  |  |
|  |  | 216 | 178 | 82,41 |  |  |
|  |  | 142 | 132 | 92,96 |  |  |
|  |  | 334 | 199 | 59,58 |  |  |
|  |  | 241 | 187 | 77,59 |  |  |
|  |  | 245 | 212 | 86,53 |  |  |
|  |  | 275 | 266 | 96,73 |  |  |
|  |  | 234 | 228 | 97,44 |  |  |
|  |  | 210 | 189 | 90,00 |  |  |
|  | ♀AfP(-) x ♂AfP(+) | 100 | 0 | 0,00 |  |  |
|  |  | 164 | 8 | 4,88 |  |  |
|  |  | 228 | 3 | 1,32 |  |  |
|  |  | 284 | 55 | 19,37 |  |  |
|  |  | 418 | 38 | 9,09 |  |  |
|  |  | 67 | 2 | 2,99 |  |  |
|  |  | 154 | 1 | 0,65 |  |  |
|  |  | 183 | 2 | 1,09 |  |  |
|  |  | 66 | 2 | 3,03 |  |  |
|  |  | 288 | 5 | 1,74 |  |  |
|  |  | 200 | 13 | 6,50 |  |  |
|  |  | 129 | 10 | 7,75 |  |  |
|  |  | 208 | 15 | 7,21 |  |  |
|  |  | 87 | 14 | 16,09 |  |  |
|  |  | 129 | 19 | 14,73 |  |  |
|  |  | 160 | 19 | 11,88 |  |  |
|  |  | 249 | 30 | 12,05 |  |  |
|  |  | 266 | 21 | 7,89 |  |  |
|  |  | 239 | 25 | 10,46 |  |  |
|  |  | 167 | 28 | 16,77 |  |  |
|  |  | 323 | 37 | 11,46 |  |  |
|  |  | 414 | 168 | 40,58 |  |  |
| Castelar | ♀AfC(+) x ♂AfC(+) | 157 | 44 | 28,03 |  |  |
|  |  | 202 | 43 | 21,29 |  |  |
|  |  | 174 | 103 | 59,20 |  |  |
|  |  | 239 | 54 | 22,59 |  |  |
|  |  | 358 | 74 | 20,67 |  |  |
|  |  | 189 | 130 | 68,78 |  |  |
|  |  | 153 | 72 | 47,06 |  |  |
|  |  | 221 | 147 | 66,52 |  |  |
|  |  | 217 | 112 | 51,61 |  |  |
|  |  | 213 | 168 | 78,87 |  |  |
|  |  | 323 | 201 | 62,23 |  |  |
|  |  | 191 | 8 | 4,19 |  |  |
|  |  | 232 | 74 | 31,90 |  |  |
|  |  | 193 | 75 | 38,86 |  |  |
|  |  | 73 | 37 | 50,68 |  |  |
|  |  | 76 | 40 | 52,63 |  |  |
|  |  | 265 | 99 | 37,36 |  |  |
|  |  | 267 | 73 | 27,34 |  |  |
|  |  | 296 | 156 | 52,70 |  |  |
|  |  | 259 | 13 | 5,02 |  |  |
|  | ♀AfC(-) x ♂AfC(-) | 149 | 4 | 2,68 |  |  |
|  |  | 192 | 76 | 39,58 |  |  |
|  |  | 176 | 49 | 27,84 |  |  |
|  |  | 183 | 85 | 46,45 |  |  |
|  |  | 165 | 103 | 62,42 |  |  |
|  |  | 194 | 3 | 1,55 |  |  |
|  |  | 99 | 34 | 34,34 |  |  |
|  |  | 96 | 33 | 34,38 |  |  |
|  |  | 213 | 43 | 20,19 |  |  |
|  |  | 166 | 82 | 49,40 |  |  |
|  |  | 96 | 6 | 6,52 |  |  |
|  |  | 132 | 8 | 6,06 |  |  |
|  |  | 337 | 77 | 22,85 |  |  |
|  |  | 198 | 126 | 63,64 |  |  |
|  |  | 186 | 36 | 19,35 |  |  |
|  | ♀AfC(+) x ♂AfC(-) | 130 | 37 | 28,46 |  |  |
|  |  | 108 | 97 | 89,81 |  |  |
|  |  | 306 | 13 | 4,25 |  |  |
|  |  | 246 | 84 | 34,15 |  |  |
|  |  | 142 | 86 | 60,56 |  |  |
|  |  | 135 | 75 | 55,56 |  |  |
|  |  | 84 | 25 | 29,76 |  |  |
|  |  | 214 | 46 | 21,50 |  |  |
|  |  | 182 | 78 | 42,86 |  |  |
|  |  | 293 | 149 | 50,85 |  |  |
|  |  | 274 | 125 | 45,62 |  |  |
|  |  | 121 | 15 | 12,40 |  |  |
|  |  | 210 | 149 | 70,95 |  |  |
|  |  | 242 | 193 | 79,75 |  |  |
|  |  | 181 | 47 | 25,97 |  |  |
|  |  | 148 | 21 | 14,19 |  |  |
|  |  | 71 | 65 | 91,55 |  |  |
|  |  | 130 | 108 | 83,08 |  |  |
|  |  | 196 | 112 | 57,14 |  |  |
|  | ♀AfC(-) x ♂AfC(+) | 239 | 1 | 0,42 |  |  |
|  |  | 154 | 7 | 4,55 |  |  |
|  |  | 180 | 30 | 16,67 |  |  |
|  |  | 200 | 39 | 19,50 |  |  |
|  |  | 94 | 24 | 25,53 |  |  |
|  |  | 278 | 7 | 2,52 |  |  |
|  |  | 70 | 5 | 7,14 |  |  |
|  |  | 103 | 18 | 17,48 |  |  |
|  |  | 115 | 10 | 8,70 |  |  |
|  |  | 237 | 38 | 16,03 |  |  |
|  |  | 187 | 14 | 7,49 |  |  |
|  |  | 176 | 34 | 19,32 |  |  |
|  |  | 109 | 41 | 37,61 |  |  |
|  |  | 230 | 85 | 36,96 |  |  |
|  |  | 228 | 69 | 30,26 |  |  |
|  |  | 108 | 3 | 2,78 |  |  |
|  |  | 61 | 17 | 27,87 |  |  |
|  |  | 59 | 18 | 30,51 |  |  |
|  |  | 237 | 4 | 1,69 |  |  |
| Peru x Castelar | ♀AfP(+) x ♂AfC(+) | 433 | 331 | 76,4 |  |  |
|  |  | 141 | 120 | 85,1 |  |  |
|  |  | 200 | 159 | 79,5 |  |  |
|  |  | 244 | 102 | 41,8 |  |  |
|  |  | 126 | 3 | 2,3 |  |  |
|  |  | 334 | 160 | 47,9 |  |  |
|  |  | 281 | 140 | 49,8 |  |  |
|  |  | 95 | 70 | 73,7 |  |  |
|  |  | 214 | 122 | 57,0 |  |  |
|  |  | 511 | 332 | 65,0 |  |  |
|  |  | 86 | 18 | 20,9 |  |  |
|  |  | 195 | 10 | 5,1 |  |  |
|  |  | 128 | 100 | 78,1 |  |  |
|  |  | 282 | 184 | 65,2 |  |  |
|  |  | 181 | 132 | 72,9 |  |  |
|  |  | 171 | 116 | 67,8 |  |  |
|  |  | 481 | 344 | 71,5 |  |  |
|  |  | 280 | 133 | 47,5 |  |  |
|  | ♀AfC(+) x ♂AfP(+) | 104 | 19 | 18,3 |  |  |
|  |  | 111 | 9 | 8,1 |  |  |
|  |  | 194 | 27 | 13,9 |  |  |
|  |  | 297 | 3 | 1,0 |  |  |
|  |  | 271 | 28 | 10,3 |  |  |
|  |  | 248 | 34 | 13,7 |  |  |
|  |  | 149 | 23 | 15,4 |  |  |
|  |  | 131 | 62 | 47,3 |  |  |
|  |  | 208 | 30 | 14,4 |  |  |
|  |  | 131 | 28 | 21,4 |  |  |
|  |  | 210 | 141 | 67,1 |  |  |
|  |  | 273 | 189 | 69,2 |  |  |
|  |  | 255 | 105 | 41,2 |  |  |
|  |  | 250 | 23 | 9,2 |  |  |
|  |  | 317 | 37 | 11,7 |  |  |
|  |  | 301 | 62 | 20,6 |  |  |
|  |  | 319 | 15 | 4,7 |  |  |
|  |  | 294 | 70 | 23,8 |  |  |
|  |  | 287 | 86 | 30,0 |  |  |
|  |  | 97 | 26 | 26,8 |  |  |
|  |  | 194 | 52 | 26,8 |  |  |
|  |  | 262 | 77 | 29,4 |  |  |
|  |  | 209 | 32 | 15,3 |  |  |
|  |  | 191 | 28 | 14,7 |  |  |
|  |  | 356 | 51 | 14,3 |  |  |
|  | ♀AfP(-) x ♂AfC(-) | 104 | 60 | 57,7 |  |  |
|  |  | 338 | 86 | 25,4 |  |  |
|  |  | 318 | 56 | 17,6 |  |  |
|  |  | 66 | 20 | 30,3 |  |  |
|  |  | 50 | 7 | 14,0 |  |  |
|  |  | 312 | 144 | 46,2 |  |  |
|  |  | 321 | 138 | 43,0 |  |  |
|  |  | 111 | 6 | 5,4 |  |  |
|  |  | 167 | 68 | 40,7 |  |  |
|  | ♀AfC(-) x ♂AfP(-) | 85 | 17 | 20,0 |  |  |
|  |  | 183 | 82 | 44,8 |  |  |
|  |  | 283 | 47 | 16,6 |  |  |
|  |  | 298 | 32 | 10,7 |  |  |
|  |  | 86 | 2 | 2,3 |  |  |
|  |  | 183 | 56 | 30,6 |  |  |
|  |  | 173 | 118 | 68,2 |  |  |
|  |  | 415 | 139 | 33,5 |  |  |
|  |  | 132 | 20 | 15,2 |  |  |
|  |  | 355 | 53 | 14,9 |  |  |
|  |  | 146 | 11 | 7,5 |  |  |
|  |  | 162 | 43 | 26,5 |  |  |
|  |  | 241 | 44 | 18,3 |  |  |
|  |  | 248 | 110 | 44,4 |  |  |
|  |  | 160 | 14 | 8,8 |  |  |
|  |  | 304 | 66 | 21,7 |  |  |
|  |  | 180 | 108 | 60,0 |  |  |
|  |  |  |  |  |  |  |
|  |  |  |  |  |  |  |
| Colony | Treatment | hatched eggs | Total pupae | % pupation |  |  |
| Peru | ♀AfP(-) x ♂AfP(-) | 42 | 0 | 0,0 |  |  |
|  |  | 19 | 0 | 0,0 |  |  |
|  |  | 22 | 1 | 4,5 |  |  |
|  |  | 12 | 9 | 75,0 |  |  |
|  |  | 25 | 18 | 72,0 |  |  |
|  |  | 47 | 25 | 53,2 |  |  |
|  |  | 120 | 45 | 37,5 |  |  |
|  |  | 99 | 50 | 50,5 |  |  |
|  |  | 94 | 51 | 54,3 |  |  |
|  |  | 67 | 58 | 86,6 |  |  |
|  |  | 94 | 62 | 66,0 |  |  |
|  |  | 96 | 62 | 64,6 |  |  |
|  |  | 122 | 68 | 55,7 |  |  |
|  |  | 54 | 0 | 0,0 |  |  |
|  |  | 148 | 0 | 0,0 |  |  |
|  |  | 152 | 90 | 59,2 |  |  |
|  |  | 208 | 111 | 53,4 |  |  |
|  |  | 207 | 0 | 0,0 |  |  |
|  |  | 230 | 154 | 67,0 |  |  |
|  |  | 322 | 187 | 58,1 |  |  |
|  | ♀AfP(+) x ♂AfP(+) | 11 | 11 | 100,0 |  |  |
|  |  | 39 | 32 | 82,1 |  |  |
|  |  | 59 | 44 | 74,6 |  |  |
|  |  | 45 | 46 | 102,2 |  |  |
|  |  | 70 | 48 | 68,6 |  |  |
|  |  | 99 | 70 | 70,7 |  |  |
|  |  | 107 | 83 | 77,6 |  |  |
|  |  | 48 | 0 | 0,0 |  |  |
|  |  | 103 | 90 | 87,4 |  |  |
|  |  | 137 | 100 | 73,0 |  |  |
|  |  | 168 | 100 | 59,5 |  |  |
|  |  | 141 | 104 | 73,8 |  |  |
|  |  | 196 | 106 | 54,1 |  |  |
|  |  | 149 | 115 | 77,2 |  |  |
|  |  | 141 | 116 | 82,3 |  |  |
|  |  | 157 | 117 | 74,5 |  |  |
|  |  | 166 | 118 | 71,1 |  |  |
|  |  | 170 | 130 | 76,5 |  |  |
|  |  | 327 | 236 | 72,2 |  |  |
|  | ♀AfP(+) x ♂AfP(-) | 29 | 17 | 58,6 |  |  |
|  |  | 59 | 36 | 61,0 |  |  |
|  |  | 81 | 47 | 58,0 |  |  |
|  |  | 41 | 52 | 126,8 |  |  |
|  |  | 96 | 60 | 62,5 |  |  |
|  |  | 81 | 68 | 84,0 |  |  |
|  |  | 107 | 88 | 82,2 |  |  |
|  |  | 106 | 92 | 86,8 |  |  |
|  |  | 153 | 101 | 66,0 |  |  |
|  |  | 166 | 103 | 62,0 |  |  |
|  |  | 194 | 107 | 55,2 |  |  |
|  |  | 178 | 109 | 61,2 |  |  |
|  |  | 132 | 110 | 83,3 |  |  |
|  |  | 199 | 110 | 55,3 |  |  |
|  |  | 212 | 149 | 70,3 |  |  |
|  |  | 266 | 150 | 56,4 |  |  |
|  |  | 228 | 154 | 67,5 |  |  |
|  |  | 189 | 157 | 83,1 |  |  |
|  | ♀AfP(-) x ♂AfP(+) | 55 | 0 | 0,0 |  |  |
|  |  | 38 | 0 | 0,0 |  |  |
|  |  | 13 | 5 | 38,5 |  |  |
|  |  | 10 | 8 | 80,0 |  |  |
|  |  | 15 | 9 | 60,0 |  |  |
|  |  | 14 | 11 | 78,6 |  |  |
|  |  | 19 | 11 | 57,9 |  |  |
|  |  | 19 | 13 | 68,4 |  |  |
|  |  | 30 | 14 | 46,7 |  |  |
|  |  | 21 | 14 | 66,7 |  |  |
|  |  | 25 | 18 | 72,0 |  |  |
|  |  | 28 | 22 | 78,6 |  |  |
|  |  | 37 | 25 | 67,6 |  |  |
|  |  | 168 | 99 | 58,9 |  |  |
| Castelar | ♀AfC(+) x ♂AfC(+) | 44 | 32 | 72,73 |  |  |
|  |  | 43 | 36 | 83,72 |  |  |
|  |  | 103 | 59 | 57,28 |  |  |
|  |  | 54 | 15 | 27,78 |  |  |
|  |  | 74 | 31 | 41,89 |  |  |
|  |  | 130 | 111 | 85,38 |  |  |
|  |  | 72 | 40 | 55,56 |  |  |
|  |  | 147 | 109 | 74,15 |  |  |
|  |  | 112 | 82 | 73,21 |  |  |
|  |  | 168 | 121 | 72,02 |  |  |
|  |  | 201 | 120 | 59,70 |  |  |
|  |  | 74 | 35 | 47,30 |  |  |
|  |  | 75 | 45 | 60,00 |  |  |
|  |  | 37 | 37 | 100,00 |  |  |
|  |  | 40 | 27 | 67,50 |  |  |
|  |  | 99 | 80 | 80,81 |  |  |
|  |  | 73 | 52 | 71,23 |  |  |
|  |  | 156 | 71 | 45,51 |  |  |
|  |  | 13 | 9 | 69,23 |  |  |
|  | ♀AfC(-) x ♂AfC(-) | 76 | 49 | 64,47 |  |  |
|  |  | 49 | 37 | 75,51 |  |  |
|  |  | 85 | 53 | 62,35 |  |  |
|  |  | 103 | 93 | 90,29 |  |  |
|  |  | 34 | 36 | 105,88 |  |  |
|  |  | 33 | 32 | 96,97 |  |  |
|  |  | 43 | 39 | 90,70 |  |  |
|  |  | 82 | 54 | 65,85 |  |  |
|  |  | 77 | 55 | 71,43 |  |  |
|  |  | 126 | 66 | 52,38 |  |  |
|  |  | 36 | 30 | 83,33 |  |  |
|  | ♀AfC(+) x ♂AfC(-) | 37 | 28 | 75,68 |  |  |
|  |  | 97 | 86 | 88,66 |  |  |
|  |  | 13 | 10 | 76,92 |  |  |
|  |  | 84 | 48 | 57,14 |  |  |
|  |  | 86 | 73 | 84,88 |  |  |
|  |  | 75 | 65 | 86,67 |  |  |
|  |  | 25 | 15 | 60,00 |  |  |
|  |  | 46 | 30 | 65,22 |  |  |
|  |  | 78 | 58 | 74,36 |  |  |
|  |  | 149 | 100 | 67,11 |  |  |
|  |  | 125 | 87 | 69,60 |  |  |
|  |  | 15 | 5 | 33,33 |  |  |
|  |  | 149 | 86 | 57,72 |  |  |
|  |  | 193 | 142 | 73,58 |  |  |
|  |  | 47 | 40 | 85,11 |  |  |
|  |  | 21 | 19 | 90,48 |  |  |
|  |  | 65 | 58 | 89,23 |  |  |
|  |  | 108 | 51 | 47,22 |  |  |
|  |  | 112 | 61 | 54,46 |  |  |
|  | ♀AfC(-) x ♂AfC(+) | 30 | 25 | 83,33 |  |  |
|  |  | 39 | 19 | 48,72 |  |  |
|  |  | 24 | 20 | 83,33 |  |  |
|  |  | 18 | 8 | 44,44 |  |  |
|  |  | 10 | 10 | 100,00 |  |  |
|  |  | 38 | 26 | 68,42 |  |  |
|  |  | 14 | 7 | 50,00 |  |  |
|  |  | 34 | 23 | 67,65 |  |  |
|  |  | 41 | 26 | 63,41 |  |  |
|  |  | 85 | 51 | 60,00 |  |  |
|  |  | 69 | 63 | 91,30 |  |  |
|  |  | 17 | 13 | 76,47 |  |  |
|  |  | 18 | 16 | 88,89 |  |  |
| Peru x Castelar | ♀AfP(+) x ♂AfC(+) | 331 | 195 | 58,9 |  |  |
|  |  | 120 | 55 | 45,8 |  |  |
|  |  | 140 | 93 | 66,4 |  |  |
|  |  | 122 | 91 | 74,6 |  |  |
|  |  | 332 | 192 | 57,8 |  |  |
|  |  | 18 | 2 | 11,1 |  |  |
|  |  | 10 | 7 | 70,0 |  |  |
|  |  | 100 | 82 | 82,0 |  |  |
|  |  | 132 | 102 | 77,3 |  |  |
|  |  | 116 | 65 | 56,0 |  |  |
|  |  | 344 | 224 | 65,1 |  |  |
|  |  | 133 | 107 | 80,5 |  |  |
|  | ♀AfC(+) x ♂AfP(+) | 19 | 13 | 68,4 |  |  |
|  |  | 27 | 24 | 88,9 |  |  |
|  |  | 28 | 26 | 92,9 |  |  |
|  |  | 34 | 26 | 76,5 |  |  |
|  |  | 23 | 19 | 82,6 |  |  |
|  |  | 62 | 58 | 93,5 |  |  |
|  |  | 30 | 25 | 83,3 |  |  |
|  |  | 28 | 13 | 46,4 |  |  |
|  |  | 141 | 113 | 80,1 |  |  |
|  |  | 189 | 154 | 81,5 |  |  |
|  |  | 105 | 78 | 74,3 |  |  |
|  |  | 23 | 20 | 87,0 |  |  |
|  |  | 37 | 31 | 83,8 |  |  |
|  |  | 62 | 51 | 82,3 |  |  |
|  |  | 15 | 5 | 33,3 |  |  |
|  |  | 70 | 50 | 71,4 |  |  |
|  |  | 86 | 61 | 70,9 |  |  |
|  |  | 26 | 24 | 92,3 |  |  |
|  |  | 52 | 31 | 59,6 |  |  |
|  |  | 32 | 22 | 68,8 |  |  |
|  |  | 28 | 25 | 89,3 |  |  |
|  |  | 51 | 47 | 92,2 |  |  |
|  | ♀AfP(-) x ♂AfC(-) | 60 | 37 | 61,7 |  |  |
|  |  | 86 | 47 | 54,7 |  |  |
|  |  | 56 | 43 | 76,8 |  |  |
|  |  | 20 | 16 | 80,0 |  |  |
|  |  | 144 | 102 | 70,8 |  |  |
|  |  | 138 | 120 | 87,0 |  |  |
|  | ♀AfC(-) x ♂AfP(-) | 17 | 10 | 58,8 |  |  |
|  |  | 82 | 55 | 67,1 |  |  |
|  |  | 47 | 31 | 66,0 |  |  |
|  |  | 32 | 31 | 96,9 |  |  |
|  |  | 56 | 38 | 67,9 |  |  |
|  |  | 118 | 75 | 63,6 |  |  |
|  |  | 139 | 87 | 62,6 |  |  |
|  |  | 53 | 37 | 69,8 |  |  |
|  |  | 11 | 9 | 81,8 |  |  |
|  |  | 44 | 26 | 59,1 |  |  |
|  |  | 110 | 60 | 54,5 |  |  |
|  |  | 14 | 9 | 64,3 |  |  |
|  |  | 108 | 82 | 75,9 |  |  |
|  |  |  |  |  |  |  |
|  |  |  |  |  |  |  |
| Colony | Treatment | Total pupae | Total emerged | % emergence |  |  |
| Peru | ♀AfP(-) x ♂AfP(-) | 18 | 16 | 88,89 |  |  |
|  |  | 25 | 25 | 100,00 |  |  |
|  |  | 45 | 44 | 97,78 |  |  |
|  |  | 50 | 50 | 100,00 |  |  |
|  |  | 51 | 51 | 100,00 |  |  |
|  |  | 58 | 58 | 100,00 |  |  |
|  |  | 62 | 62 | 100,00 |  |  |
|  |  | 62 | 57 | 91,94 |  |  |
|  |  | 68 | 68 | 100,00 |  |  |
|  |  | 90 | 89 | 98,89 |  |  |
|  |  | 111 | 89 | 80,18 |  |  |
|  |  | 154 | 152 | 98,70 |  |  |
|  |  | 187 | 155 | 82,89 |  |  |
|  | ♀AfP(+) x ♂AfP(+) | 11 | 11 | 100,00 |  |  |
|  |  | 32 | 32 | 100,00 |  |  |
|  |  | 44 | 44 | 100,00 |  |  |
|  |  | 46 | 46 | 100,00 |  |  |
|  |  | 48 | 47 | 97,92 |  |  |
|  |  | 70 | 69 | 98,57 |  |  |
|  |  | 83 | 81 | 97,59 |  |  |
|  |  | 90 | 88 | 97,78 |  |  |
|  |  | 100 | 99 | 99,00 |  |  |
|  |  | 100 | 100 | 100,00 |  |  |
|  |  | 104 | 104 | 100,00 |  |  |
|  |  | 106 | 103 | 97,17 |  |  |
|  |  | 115 | 114 | 99,13 |  |  |
|  |  | 116 | 116 | 100,00 |  |  |
|  |  | 117 | 117 | 100,00 |  |  |
|  |  | 118 | 118 | 100,00 |  |  |
|  |  | 130 | 130 | 100,00 |  |  |
|  |  | 236 | 235 | 99,58 |  |  |
|  | ♀AfP(+) x ♂AfP(-) | 17 | 14 | 82,35 |  |  |
|  |  | 36 | 36 | 100,00 |  |  |
|  |  | 47 | 45 | 95,74 |  |  |
|  |  | 52 | 52 | 100,00 |  |  |
|  |  | 60 | 60 | 100,00 |  |  |
|  |  | 68 | 68 | 100,00 |  |  |
|  |  | 88 | 88 | 100,00 |  |  |
|  |  | 92 | 92 | 100,00 |  |  |
|  |  | 101 | 100 | 99,01 |  |  |
|  |  | 103 | 102 | 99,03 |  |  |
|  |  | 107 | 106 | 99,07 |  |  |
|  |  | 109 | 107 | 98,17 |  |  |
|  |  | 110 | 109 | 99,09 |  |  |
|  |  | 110 | 107 | 97,27 |  |  |
|  |  | 149 | 149 | 100,00 |  |  |
|  |  | 150 | 150 | 100,00 |  |  |
|  |  | 154 | 154 | 100,00 |  |  |
|  |  | 157 | 157 | 100,00 |  |  |
|  | ♀AfP(-) x ♂AfP(+) | 11 | 11 | 100,00 |  |  |
|  |  | 11 | 11 | 100,00 |  |  |
|  |  | 13 | 13 | 100,00 |  |  |
|  |  | 14 | 14 | 100,00 |  |  |
|  |  | 14 | 14 | 100,00 |  |  |
|  |  | 18 | 16 | 88,89 |  |  |
|  |  | 22 | 19 | 86,36 |  |  |
|  |  | 25 | 24 | 96,00 |  |  |
|  |  | 99 | 90 | 90,91 |  |  |
| Castelar | ♀AfC(+) x ♂AfC(+) | 32 | 32 | 100,00 |  |  |
|  |  | 36 | 36 | 100,00 |  |  |
|  |  | 59 | 57 | 96,61 |  |  |
|  |  | 15 | 14 | 93,33 |  |  |
|  |  | 31 | 30 | 96,77 |  |  |
|  |  | 111 | 110 | 99,10 |  |  |
|  |  | 40 | 39 | 97,50 |  |  |
|  |  | 109 | 107 | 98,17 |  |  |
|  |  | 82 | 82 | 100,00 |  |  |
|  |  | 121 | 106 | 87,60 |  |  |
|  |  | 120 | 118 | 98,33 |  |  |
|  |  | 35 | 34 | 97,14 |  |  |
|  |  | 45 | 43 | 95,56 |  |  |
|  |  | 37 | 37 | 100,00 |  |  |
|  |  | 27 | 27 | 100,00 |  |  |
|  |  | 80 | 77 | 96,25 |  |  |
|  |  | 52 | 52 | 100,00 |  |  |
|  |  | 71 | 70 | 98,59 |  |  |
|  | ♀AfC(-) x ♂AfC(-) | 49 | 47 | 95,92 |  |  |
|  |  | 37 | 36 | 97,30 |  |  |
|  |  | 53 | 53 | 100,00 |  |  |
|  |  | 93 | 92 | 98,92 |  |  |
|  |  | 36 | 36 | 100,00 |  |  |
|  |  | 32 | 32 | 100,00 |  |  |
|  |  | 39 | 38 | 97,44 |  |  |
|  |  | 54 | 53 | 98,15 |  |  |
|  |  | 55 | 55 | 100,00 |  |  |
|  |  | 66 | 66 | 100,00 |  |  |
|  |  | 30 | 30 | 100,00 |  |  |
|  | ♀AfC(+) x ♂AfC(-) | 28 | 26 | 92,86 |  |  |
|  |  | 86 | 84 | 97,67 |  |  |
|  |  | 10 | 10 | 100,00 |  |  |
|  |  | 48 | 48 | 100,00 |  |  |
|  |  | 73 | 62 | 84,93 |  |  |
|  |  | 65 | 64 | 98,46 |  |  |
|  |  | 15 | 14 | 93,33 |  |  |
|  |  | 30 | 28 | 93,33 |  |  |
|  |  | 58 | 57 | 98,28 |  |  |
|  |  | 100 | 100 | 100,00 |  |  |
|  |  | 87 | 84 | 96,55 |  |  |
|  |  | 86 | 74 | 86,05 |  |  |
|  |  | 142 | 139 | 97,89 |  |  |
|  |  | 40 | 39 | 97,50 |  |  |
|  |  | 19 | 19 | 100,00 |  |  |
|  |  | 58 | 56 | 96,55 |  |  |
|  |  | 51 | 49 | 96,08 |  |  |
|  |  | 61 | 56 | 91,80 |  |  |
|  | ♀AfC(-) x ♂AfC(+) | 25 | 25 | 100,00 |  |  |
|  |  | 19 | 19 | 100,00 |  |  |
|  |  | 20 | 19 | 95,00 |  |  |
|  |  | 10 | 10 | 100,00 |  |  |
|  |  | 26 | 26 | 100,00 |  |  |
|  |  | 23 | 22 | 95,65 |  |  |
|  |  | 26 | 24 | 92,31 |  |  |
|  |  | 51 | 46 | 90,20 |  |  |
|  |  | 63 | 58 | 92,06 |  |  |
|  |  | 13 | 13 | 100,00 |  |  |
|  |  | 16 | 15 | 93,75 |  |  |
| Peru x Castelar | ♀AfP(+) x ♂AfC(+) | 195 | 195 | 100,00 |  |  |
|  |  | 55 | 54 | 98,18 |  |  |
|  |  | 93 | 93 | 100,00 |  |  |
|  |  | 91 | 91 | 100,00 |  |  |
|  |  | 192 | 192 | 100,00 |  |  |
|  |  | 82 | 82 | 100,00 |  |  |
|  |  | 102 | 102 | 100,00 |  |  |
|  |  | 65 | 65 | 100,00 |  |  |
|  |  | 224 | 224 | 100,00 |  |  |
|  |  | 107 | 107 | 100,00 |  |  |
|  | ♀AfC(+) x ♂AfP(+) | 13 | 13 | 100,00 |  |  |
|  |  | 24 | 24 | 100,00 |  |  |
|  |  | 26 | 26 | 100,00 |  |  |
|  |  | 26 | 26 | 100,00 |  |  |
|  |  | 19 | 19 | 100,00 |  |  |
|  |  | 58 | 58 | 100,00 |  |  |
|  |  | 25 | 25 | 100,00 |  |  |
|  |  | 13 | 13 | 100,00 |  |  |
|  |  | 113 | 113 | 100,00 |  |  |
|  |  | 154 | 154 | 100,00 |  |  |
|  |  | 78 | 78 | 100,00 |  |  |
|  |  | 20 | 20 | 100,00 |  |  |
|  |  | 31 | 31 | 100,00 |  |  |
|  |  | 51 | 51 | 100,00 |  |  |
|  |  | 50 | 50 | 100,00 |  |  |
|  |  | 61 | 61 | 100,00 |  |  |
|  |  | 24 | 24 | 100,00 |  |  |
|  |  | 31 | 31 | 100,00 |  |  |
|  |  | 22 | 22 | 100,00 |  |  |
|  |  | 25 | 23 | 92,00 |  |  |
|  |  | 47 | 47 | 100,00 |  |  |
|  | ♀AfP(-) x ♂AfC(-) | 37 | 37 | 100,00 |  |  |
|  |  | 47 | 47 | 100,00 |  |  |
|  |  | 43 | 43 | 100,00 |  |  |
|  |  | 16 | 16 | 100,00 |  |  |
|  |  | 102 | 102 | 100,00 |  |  |
|  |  | 120 | 120 | 100,00 |  |  |
|  | ♀AfC(-) x ♂AfP(-) | 10 | 10 | 100,00 |  |  |
|  |  | 55 | 55 | 100,00 |  |  |
|  |  | 31 | 31 | 100,00 |  |  |
|  |  | 31 | 31 | 100,00 |  |  |
|  |  | 38 | 38 | 100,00 |  |  |
|  |  | 75 | 75 | 100,00 |  |  |
|  |  | 87 | 85 | 97,70 |  |  |
|  |  | 37 | 37 | 100,00 |  |  |
|  |  | 26 | 26 | 100,00 |  |  |
|  |  | 60 | 58 | 96,67 |  |  |
|  |  | 82 | 82 | 100,00 |  |  |
|  |  |  |  |  |  |  |
|  |  |  |  |  |  |  |
| Colony | Treatment | Females | Males | Non emerged | Total pupae | Sex ratio |
| Peru | ♀AfP(-) x ♂AfP(-) | 22 | 40 | 0 | 62 | 0,35 |
|  |  | 34 | 55 | 22 | 111 | 0,38 |
|  |  | 20 | 29 | 0 | 50 | 0,41 |
|  |  | 29 | 39 | 0 | 68 | 0,43 |
|  |  | 12 | 13 | 0 | 25 | 0,48 |
|  |  | 43 | 46 | 1 | 90 | 0,48 |
|  |  | 8 | 8 | 2 | 18 | 0,50 |
|  |  | 76 | 76 | 2 | 154 | 0,50 |
|  |  | 76 | 76 | 32 | 187 | 0,50 |
|  |  | 22 | 21 | 1 | 45 | 0,51 |
|  |  | 30 | 28 | 0 | 58 | 0,52 |
|  |  | 30 | 27 | 5 | 62 | 0,53 |
|  |  | 26 | 21 | 0 | 51 | 0,55 |
|  | ♀AfP(+) x ♂AfP(+) | 40 | 77 | 0 | 117 | 0,34 |
|  |  | 41 | 72 | 1 | 115 | 0,36 |
|  |  | 19 | 25 | 0 | 44 | 0,43 |
|  |  | 58 | 72 | 0 | 130 | 0,45 |
|  |  | 21 | 26 | 1 | 48 | 0,45 |
|  |  | 53 | 65 | 0 | 118 | 0,45 |
|  |  | 31 | 38 | 1 | 70 | 0,45 |
|  |  | 107 | 128 | 1 | 236 | 0,46 |
|  |  | 46 | 53 | 0 | 100 | 0,46 |
|  |  | 54 | 62 | 0 | 116 | 0,47 |
|  |  | 15 | 17 | 0 | 32 | 0,47 |
|  |  | 49 | 55 | 0 | 104 | 0,47 |
|  |  | 50 | 49 | 1 | 100 | 0,51 |
|  |  | 45 | 43 | 2 | 90 | 0,51 |
|  |  | 43 | 38 | 2 | 83 | 0,53 |
|  |  | 6 | 5 | 0 | 11 | 0,55 |
|  |  | 59 | 44 | 3 | 106 | 0,57 |
|  |  | 29 | 17 | 0 | 46 | 0,63 |
|  | ♀AfP(+) x ♂AfP(-) | 33 | 67 | 1 | 101 | 0,33 |
|  |  | 39 | 68 | 2 | 109 | 0,36 |
|  |  | 6 | 8 | 3 | 17 | 0,43 |
|  |  | 50 | 59 | 1 | 110 | 0,46 |
|  |  | 48 | 54 | 1 | 103 | 0,47 |
|  |  | 17 | 19 | 0 | 36 | 0,47 |
|  |  | 73 | 76 | 0 | 149 | 0,49 |
|  |  | 53 | 54 | 3 | 110 | 0,50 |
|  |  | 35 | 33 | 0 | 68 | 0,51 |
|  |  | 80 | 74 | 0 | 154 | 0,52 |
|  |  | 46 | 42 | 0 | 88 | 0,52 |
|  |  | 32 | 28 | 0 | 60 | 0,53 |
|  |  | 80 | 70 | 0 | 150 | 0,53 |
|  |  | 84 | 72 | 0 | 157 | 0,54 |
|  |  | 51 | 41 | 0 | 92 | 0,55 |
|  |  | 25 | 20 | 2 | 47 | 0,56 |
|  |  | 62 | 44 | 1 | 107 | 0,58 |
|  |  | 32 | 20 | 0 | 52 | 0,62 |
|  | ♀AfP(-) x ♂AfP(+) | 5 | 13 | 3 | 22 | 0,26 |
|  |  | 4 | 9 | 0 | 13 | 0,31 |
|  |  | 5 | 9 | 0 | 14 | 0,36 |
|  |  | 9 | 14 | 1 | 25 | 0,42 |
|  |  | 7 | 9 | 2 | 18 | 0,44 |
|  |  | 40 | 48 | 9 | 99 | 0,46 |
|  |  | 8 | 6 | 0 | 14 | 0,57 |
|  |  | 8 | 3 | 0 | 11 | 0,73 |
|  |  | 8 | 3 | 0 | 11 | 0,73 |
| Castelar | ♀AfC(+) x ♂AfC(+) | 11 | 21 | 0 | 32 | 0,34 |
|  |  | 13 | 23 | 0 | 37 | 0,35 |
|  |  | 14 | 20 | 1 | 35 | 0,41 |
|  |  | 52 | 55 | 2 | 109 | 0,49 |
|  |  | 14 | 14 | 1 | 31 | 0,50 |
|  |  | 38 | 38 | 3 | 80 | 0,50 |
|  |  | 26 | 26 | 0 | 52 | 0,50 |
|  |  | 29 | 28 | 2 | 59 | 0,51 |
|  |  | 22 | 21 | 2 | 45 | 0,51 |
|  |  | 62 | 56 | 2 | 120 | 0,53 |
|  |  | 17 | 15 | 0 | 36 | 0,53 |
|  |  | 57 | 48 | 15 | 121 | 0,54 |
|  |  | 38 | 32 | 1 | 71 | 0,54 |
|  |  | 61 | 49 | 1 | 111 | 0,55 |
|  |  | 15 | 12 | 0 | 27 | 0,56 |
|  |  | 47 | 35 | 0 | 82 | 0,57 |
|  |  | 9 | 5 | 1 | 15 | 0,64 |
|  |  | 26 | 13 | 1 | 40 | 0,67 |
|  | ♀AfC(-) x ♂AfC(-) | 20 | 33 | 0 | 53 | 0,38 |
|  |  | 20 | 33 | 1 | 54 | 0,38 |
|  |  | 20 | 33 | 0 | 55 | 0,38 |
|  |  | 12 | 18 | 0 | 30 | 0,40 |
|  |  | 12 | 19 | 0 | 32 | 0,41 |
|  |  | 26 | 38 | 0 | 66 | 0,41 |
|  |  | 15 | 21 | 0 | 36 | 0,42 |
|  |  | 16 | 19 | 1 | 37 | 0,47 |
|  |  | 47 | 45 | 1 | 93 | 0,51 |
|  |  | 28 | 18 | 2 | 49 | 0,60 |
|  |  | 27 | 11 | 1 | 39 | 0,71 |
|  | ♀AfC(+) x ♂AfC(-) | 8 | 11 | 0 | 19 | 0,42 |
|  |  | 21 | 27 | 0 | 48 | 0,44 |
|  |  | 39 | 45 | 2 | 86 | 0,46 |
|  |  | 27 | 30 | 1 | 58 | 0,47 |
|  |  | 48 | 51 | 0 | 100 | 0,48 |
|  |  | 41 | 43 | 3 | 87 | 0,49 |
|  |  | 31 | 31 | 11 | 73 | 0,50 |
|  |  | 25 | 24 | 2 | 51 | 0,51 |
|  |  | 38 | 36 | 12 | 86 | 0,51 |
|  |  | 13 | 12 | 2 | 28 | 0,52 |
|  |  | 33 | 29 | 1 | 65 | 0,53 |
|  |  | 30 | 26 | 5 | 61 | 0,54 |
|  |  | 31 | 25 | 2 | 58 | 0,55 |
|  |  | 78 | 60 | 3 | 142 | 0,57 |
|  |  | 16 | 12 | 2 | 30 | 0,57 |
|  |  | 29 | 10 | 1 | 40 | 0,74 |
|  |  | 11 | 3 | 1 | 15 | 0,79 |
|  |  | 8 | 2 | 0 | 10 | 0,80 |
|  | ♀AfC(-) x ♂AfC(+) | 3 | 7 | 0 | 10 | 0,30 |
|  |  | 8 | 17 | 0 | 25 | 0,32 |
|  |  | 6 | 9 | 1 | 16 | 0,40 |
|  |  | 10 | 14 | 2 | 26 | 0,42 |
|  |  | 8 | 11 | 1 | 20 | 0,42 |
|  |  | 25 | 33 | 5 | 63 | 0,43 |
|  |  | 11 | 11 | 1 | 23 | 0,50 |
|  |  | 24 | 22 | 5 | 51 | 0,52 |
|  |  | 10 | 9 | 0 | 19 | 0,53 |
|  |  | 7 | 6 | 0 | 13 | 0,54 |
|  |  | 16 | 10 | 0 | 26 | 0,62 |
| Peru x Castelar | ♀AfP(+) x ♂AfC(+) | 20 | 34 | 1 | 55 | 0,37 |
|  |  | 93 | 99 | 0 | 192 | 0,48 |
|  |  | 40 | 42 | 0 | 82 | 0,49 |
|  |  | 46 | 45 | 0 | 91 | 0,51 |
|  |  | 100 | 95 | 0 | 195 | 0,51 |
|  |  | 35 | 30 | 0 | 65 | 0,54 |
|  |  | 51 | 42 | 0 | 93 | 0,55 |
|  |  | 62 | 45 | 0 | 107 | 0,58 |
|  |  | 61 | 41 | 0 | 102 | 0,60 |
|  |  | 135 | 89 | 0 | 224 | 0,60 |
|  | ♀AfC(+) x ♂AfP(+) | 3 | 10 | 0 | 13 | 0,23 |
|  |  | 8 | 16 | 0 | 24 | 0,33 |
|  |  | 8 | 12 | 0 | 20 | 0,40 |
|  |  | 13 | 18 | 0 | 31 | 0,42 |
|  |  | 8 | 11 | 0 | 19 | 0,42 |
|  |  | 11 | 15 | 0 | 26 | 0,42 |
|  |  | 14 | 17 | 0 | 31 | 0,45 |
|  |  | 11 | 12 | 1 | 25 | 0,48 |
|  |  | 12 | 13 | 0 | 25 | 0,48 |
|  |  | 74 | 80 | 0 | 154 | 0,48 |
|  |  | 23 | 24 | 0 | 47 | 0,49 |
|  |  | 25 | 26 | 0 | 51 | 0,49 |
|  |  | 13 | 13 | 0 | 26 | 0,50 |
|  |  | 39 | 39 | 0 | 78 | 0,50 |
|  |  | 25 | 25 | 0 | 50 | 0,50 |
|  |  | 11 | 11 | 0 | 22 | 0,50 |
|  |  | 30 | 28 | 0 | 58 | 0,52 |
|  |  | 13 | 11 | 0 | 24 | 0,54 |
|  |  | 63 | 50 | 0 | 113 | 0,56 |
|  |  | 37 | 24 | 0 | 61 | 0,61 |
|  |  | 8 | 5 | 0 | 13 | 0,62 |
|  | ♀AfP(-) x ♂AfC(-) | 14 | 23 | 0 | 37 | 0,38 |
|  |  | 21 | 26 | 0 | 47 | 0,45 |
|  |  | 20 | 23 | 0 | 43 | 0,47 |
|  |  | 10 | 10 | 0 | 20 | 0,50 |
|  |  | 69 | 51 | 0 | 120 | 0,58 |
|  |  | 60 | 42 | 0 | 102 | 0,59 |
|  | ♀AfC(-) x ♂AfP(-) | 11 | 20 | 0 | 31 | 0,35 |
|  |  | 14 | 23 | 0 | 37 | 0,38 |
|  |  | 12 | 14 | 0 | 26 | 0,46 |
|  |  | 19 | 19 | 0 | 38 | 0,50 |
|  |  | 43 | 42 | 2 | 87 | 0,51 |
|  |  | 38 | 37 | 0 | 75 | 0,51 |
|  |  | 30 | 28 | 0 | 60 | 0,52 |
|  |  | 29 | 26 | 0 | 55 | 0,53 |
|  |  | 44 | 38 | 0 | 83 | 0,54 |
|  |  | 17 | 14 | 0 | 31 | 0,55 |
|  |  | 6 | 4 | 0 | 10 | 0,60 |

**Supplementary Figure 1.** PCR amplification using the *Wolbachia*-specific primers wspecF/R for the 16S rRNA gene of the colonies of *Anastrepha fraterculus* Brazilian-1 (AfC) and Peruvian (AfP) morphotypes, both naturally infected with *Wolbachia*. This reaction is expected to amplify a 438 bp fragment.


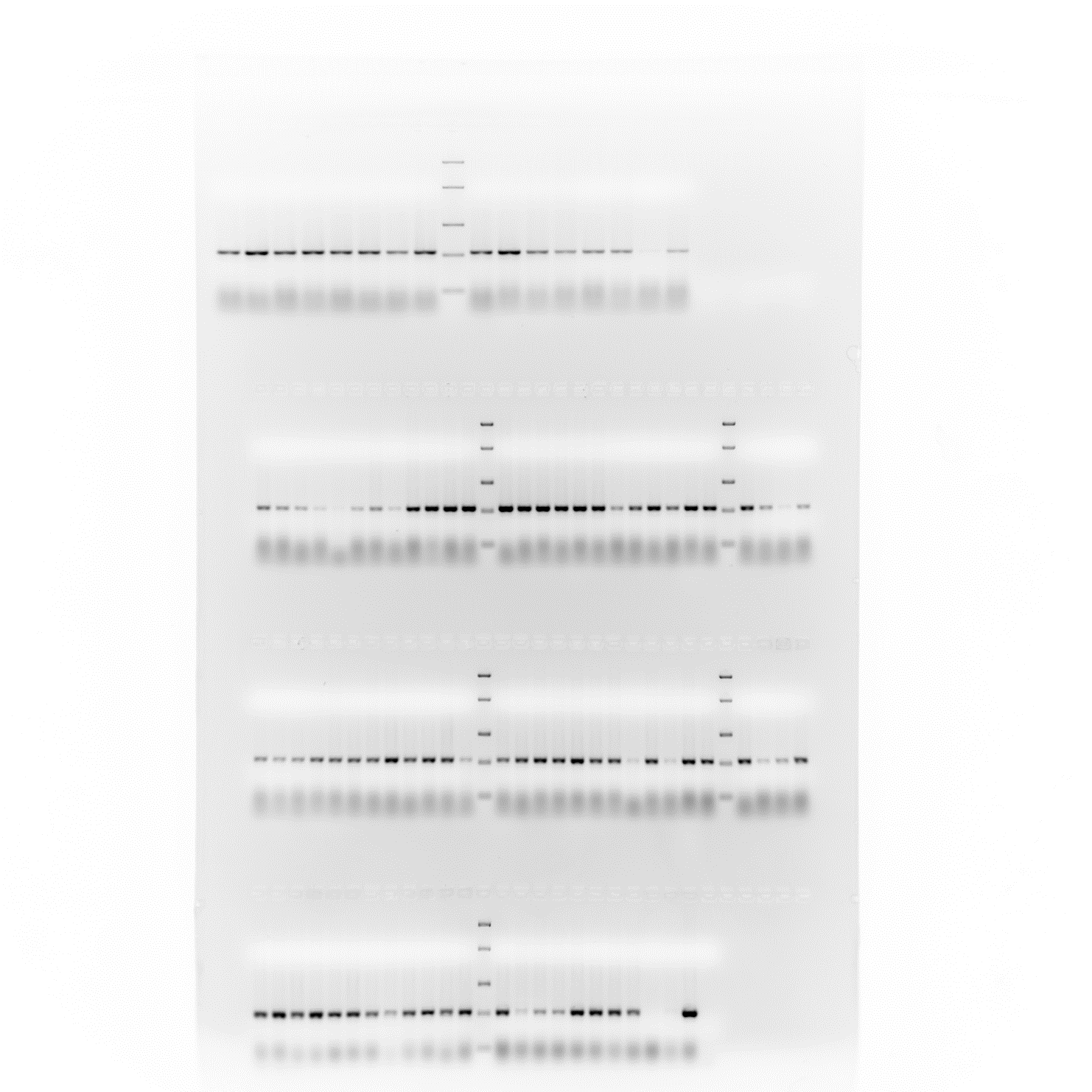


+ -

*

*

*

**

**


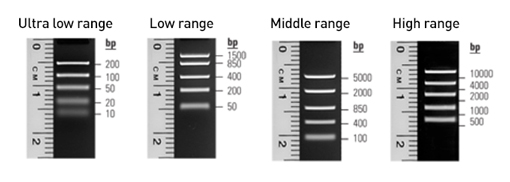


L

L

L

L

*: twenty individuals from the *A. fraterculus* Peruvian morphotype.

**: twenty individuals from the *A. fraterculus* Brazilian-1 morphotype.

+: positive control (wCer2 from the ’88.6’ *C. capitata* transinfected strain).

-: PCR negative control.

L: The FastRuler Middle Range DNA Ladder was used. Expected bands are shown at the bottom right

corner of the figure.

**Supplementary Figure 2.** PCR amplification using the *Wolbachia*-specific primers wspecF/R for the 16S rRNA gene of *Anastrepha fraterculus* Peruvian morphotype (AfP) treated with 0.1% tetracycline. This reaction is expected to amplify a 438 bp fragment.


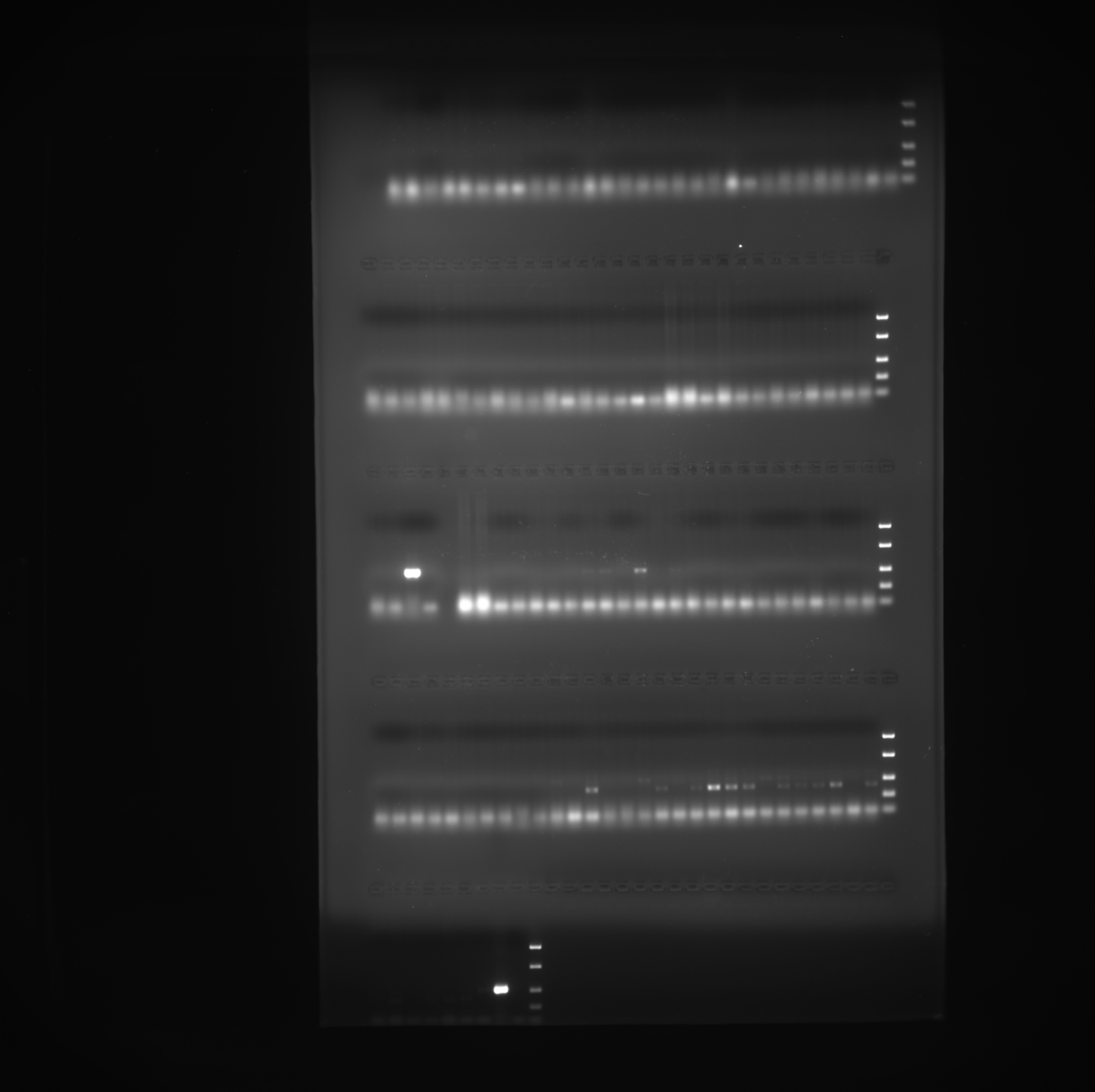

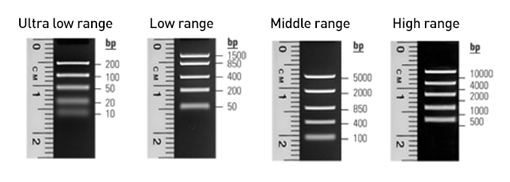


+

-

*

*

*

L

L

*: twenty individuals from the tetracycline-treated *A. fraterculus* Peruvian morphotype;

+: positive control (wCer2 from the ‘88.6’ *C. capitata* transinfected strain);

-: negative control for the PCR reaction

L: The FastRuler Low Range DNA Ladder was used. Expected bands are shown at the right bottom

corner of the Figure.

**Supplementary Figure 3.** PCR amplification using the *Wolbachia*-specific primers wspecF/R for the 16S rRNA gene of *Anastrepha fraterculus* Brazilian-1 morphotype (AfC) treated with 0.01% rifampicin. This reaction is expected to amplify a 438 bp fragment.


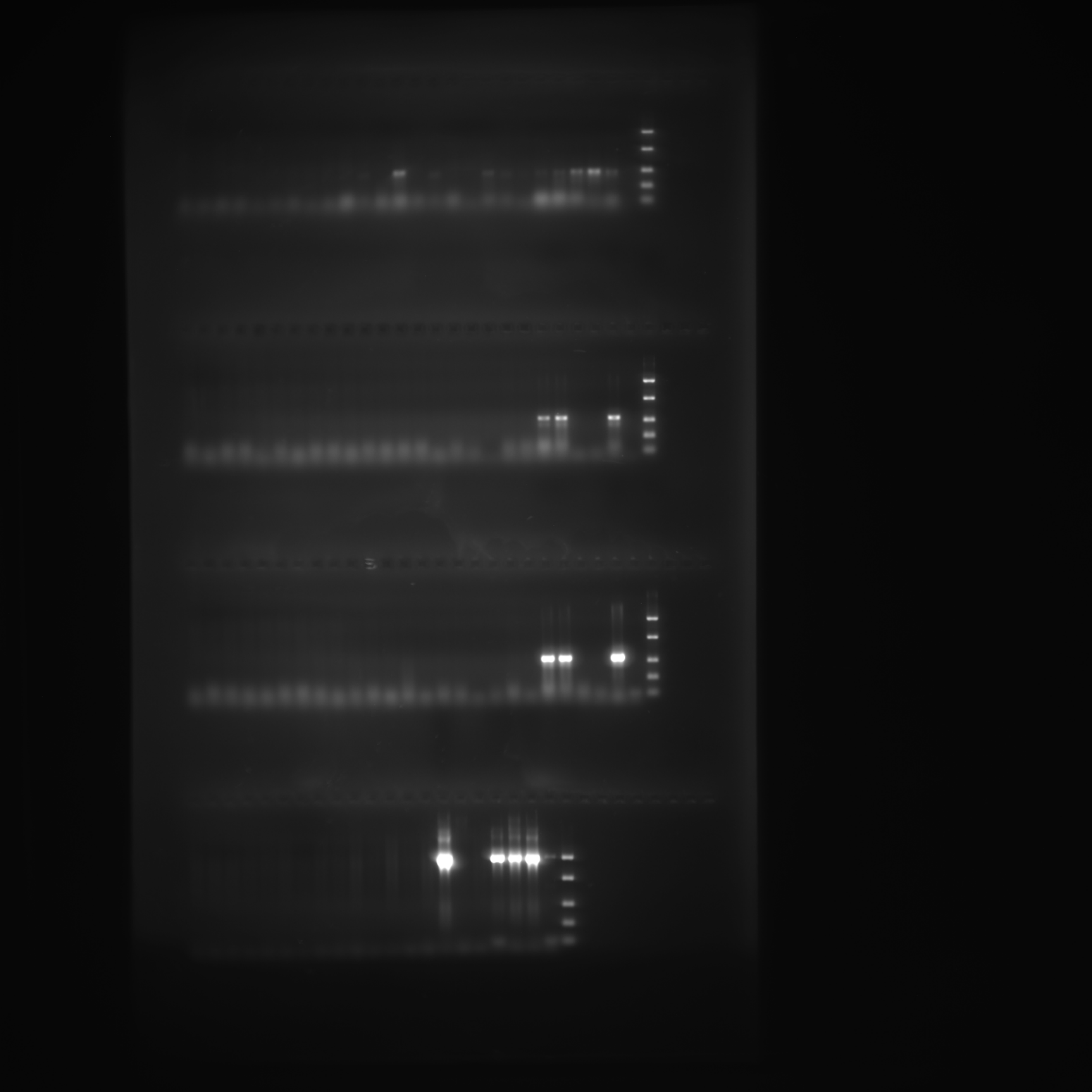

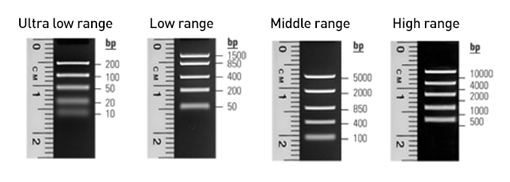


+^1^

+^2^

+^3^

- -

*

L

*: twenty individuals from the rifampicin-treated *A. fraterculus* Brazilian morphotype.

+1: positive control (wCer2 from the ‘88.6’ *C. capitata* transinfected strain).

+2: positive control (wCer4 from the ‘S.10.3’ *C. capitata* transinfected strain).

-: negative control for DNA extraction and PCR reaction.

+3: positive control (from the naturally infected *A. fraterculus* Brazilian morphotype).

L: The FastRuler Low Range DNA Ladder was used. Expected bands are shown at the right bottom

corner of the Figure.
